# Enrichment and Aggregation of Purple Non-sulfur Bacteria in a Mixed-Culture Sequencing-Batch Photobioreactor for Biological Nutrient Removal From Wastewater

**DOI:** 10.3389/fbioe.2020.557234

**Published:** 2020-12-17

**Authors:** Marta Cerruti, Berber Stevens, Sirous Ebrahimi, Abbas Alloul, Siegfried E. Vlaeminck, David G. Weissbrodt

**Affiliations:** ^1^Department of Biotechnology, Delft University of Technology, Delft, Netherlands; ^2^Department of Chemical Engineering, Sahand University of Technology, Tabriz, Iran; ^3^Department of Bioscience Engineering, University of Antwerp, Antwerp, Belgium

**Keywords:** mixed-culture biotechnology, environmental biotechnology, microbial ecology, sequencing batch reactor, bioaggregation, purple phototrophic bacteria, resource recovery, biological wastewater treatment

## Abstract

Mixed-culture biotechnologies are widely used to capture nutrients from wastewater. Purple non-sulfur bacteria (PNSB), a guild of anoxygenic photomixotrophic organisms, rise interest for their ability to directly assimilate nutrients in the biomass. One challenge targets the aggregation and accumulation of PNSB biomass to separate it from the treated water. Our aim was to enrich and produce a concentrated, fast-settling PNSB biomass with high nutrient removal capacity in a 1.5-L, stirred-tank, anaerobic sequencing-batch photobioreactor (SBR). PNSB were rapidly enriched after inoculation with activated sludge at 0.1 gVSS L^–1^ in a first batch of 24 h under continuous irradiance of infrared (IR) light (>700 nm) at 375 W m^–2^, with *Rhodobacter* reaching 54% of amplicon sequencing read counts. SBR operations with decreasing hydraulic retention times (48 to 16 h, i.e., 1–3 cycles d^–1^) and increasing volumetric organic loading rates (0.2–1.3 kg COD d^–1^ m^–3^) stimulated biomass aggregation, settling, and accumulation in the system, reaching as high as 3.8 g VSS L^–1^. The sludge retention time (SRT) increased freely from 2.5 to 11 days. Acetate, ammonium, and orthophosphate were removed up to 96% at a rate of 1.1 kg COD d^–1^ m^–3^, 77% at 113 g N d^–1^ m^–3^, and 73% at 15 g P d^–1^ m^–3^, respectively, with COD:N:P assimilation ratio of 100:6.7:0.9 m/m/m. SBR regime shifts sequentially selected for *Rhodobacter* (90%) under shorter SRT and non-limiting concentration of acetate during reaction phases, for *Rhodopseudomonas* (70%) under longer SRT and acetate limitation during reaction, and *Blastochloris* (10%) under higher biomass concentrations, underlying competition for substrate and photons in the PNSB guild. With SBR operations we produced a fast-settling biomass, highly (>90%) enriched in PNSB. A high nutrient removal was achieved by biomass assimilation, reaching the European nutrient discharge limits. We opened further insights on the microbial ecology of PNSB-based processes for water resource recovery.

## Graphical Abstract

Sequencing batch reactor enrichment and aggregation of purple non-sulfur bacteria.

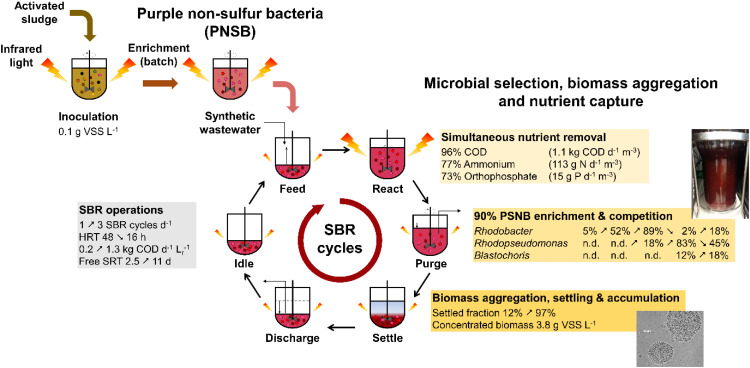

## Introduction

Biological nutrient removal (BNR) is one of the main goals of wastewater treatment to safeguard aquatic ecosystems from anoxia and eutrophication. Water quality regulations become stricter on the limits of nutrient discharge and removal. European quality criteria target the following residual concentrations and removal of organic matter (125 mg COD_Tot_ L^–1^ and 75% removal; 25 mg BOD_5_ L^–1^ and 70–90% removal), nitrogen (10–15 mg N_Tot_ L^–1^, 70–80% removal), and phosphorus (1–2 mg P_Tot_ L^–1^, 80% removal) (EUR-Lex, 1991; Guimarães et al., 2018). Besides conventional activated sludge systems, research and innovation target the use of novel microbial processes for water resource recovery (Guest et al., 2009; Verstraete and Vlaeminck, 2011; Alloul et al., 2018) on top of pollution control.

In the resource recovery context, purple non-sulfur bacteria (PNSB) can propel a sustainable treatment by capturing nutrient resource from used water (Verstraete et al., 2016; Puyol et al., 2017b), valorizing waste into biomass, bioenergy, bulk chemicals, and biomaterials. PNSB form an attractive guild of phototrophic organisms with a facultative anaerobic and hyperversatile metabolism that allows them to grow under ever-changing environmental conditions (van Niel, 1944; Imhoff, 2017). They populate the surface of aquatic environments by absorbing sunlight at different wavelengths, using carotenoids (absorbing in the visible spectrum) and bacteriochlorophylls (absorbing the infrared light, IR, at wavelengths above 700 nm), with a competitive advantage in a mixed-culture microbial ecosystem. PNSB can switch between photoorganoheterotrophy, photolithoautotrophy, respiratory or fermentative chemoorgano heterotrophy, respiratory chemolithoautotrophy, and nitrogen fixation depending on the composition of electron donors and acceptors present in their surrounding (Madigan and Jung, 2009). This enables them to thrive on different pools of electron donors, recycle electrons, achieve redox homeostasis, and grow under alternation of light and dark (McEwan, 1994). PNSB ferment reduced organics into carboxylates in the dark, photoferment them into dihydrogen, or accumulate and condense them as intracellular storage polymers like biopolyesters (e.g., poly-β-hydroxyalkanoates, PHAs) as electron sinks under nutrient limitations (Hustede et al., 1993). Rediscovering PNSB for ecotechnologies and nutrient capture goes via basic study of their metabolism and selection features from pure to mixed cultures, and eco-design to develop robust, non-axenic, and economically appealing processes (Bryant and Frigaard, 2006).

The potential of PNSB for converting diverse carbon sources such as volatile fatty acids (acetate, malate, butyrate and propionate), sugars or alcohols, has been screened with isolates (Stoppani et al., 1955; Madigan and Gest, 1979; Alloul et al., 2019), underlying the potential of populations of this guild for water treatment. PNSB can assimilate carbon (C), nitrogen (N), and phosphorus (P) from wastewater at COD:N:P ratio of 100:7:2 versus 100:5:1 m/m/m for activated sludge, with an elemental formula for purple phototrophic biomass given as C_1_H_1_._8_O_0_._38_N_0_._18_ (degree of reduction of 4.5 mol e^–^ C-mol^–1^ X_PPB_) (Puyol et al., 2017a). Their photon-capturing and energy-recycling physiology leads PNSB to achieve rapid biomass specific maximum growth rates (μ_max_) of 1.7–5.3 d^–1^ and biomass yields (Y_X/COD_) on organic substrates (expressed as chemical oxygen demand, COD) of 0.6–1.2 g COD_X_ g^–1^ COD_S_ from mixed to pure cultures (Eroglu et al., 1999; Hülsen et al., 2014), involving additional electron sources from the bulk liquid phase. New-generation biological wastewater treatment processes aim to decrease sludge production and handling, by making use of slow-growing and low-yield microorganisms such as polyphosphate-accumulating and anammox bacteria. In contrast, the use of organisms with a high biomass yield such as PNSB is of definite interest to remove, capture and concentrate carbon, nitrogen and phosphorus nutrient resources out of the wastewater by assimilation into the biomass. The biomass can be then valorized to generate energy through methanization and to produce, e.g., single-cell proteins (i.e., source of microbial proteins), bioplastics via PHAs, and biohydrogen on concentrated streams (Honda et al., 2006; Puyol et al., 2017b).

Technically, one important challenge of photobiotechnologies resides in the limitation of photon supply across the reactor bulk (Pulz, 2001), therefore, many PNSB-based processes have been operated at concentrations below 1 g VSS L^–1^. Light limitation is often considered *a priori* as a killing factor for the process performance and economics, while such low biomass concentration can remain a drawback for the intensification of volumetric conversions.

Mixed-culture processes are actively investigated to harness the ability of PNSB to treat wastewater (Nakajima et al., 1997; Hülsen et al., 2016; Verstraete et al., 2016), starting from stabilization ponds (Freedman et al., 1983; Almasi and Pescod, 1996). Process configurations involved continuous up-flow system (Driessens et al., 1987), continuous-flow stirred tank reactor (Alloul et al., 2019), tubular reactor (Carlozzi et al., 2006), sequencing batch reactor (SBR) (Chitapornpan et al., 2012; Fradinho et al., 2013), membrane bioreactor (MBR) (Hülsen et al., 2016), and membrane sequencing batch reactor (MSBR) (Kaewsuk et al., 2010). One challenge in the application of PNSB organisms is considered to remain in the solid-liquid (S/L) separation of the biomass from the aqueous stream. Decoupling the hydraulic (HRT) and solid (SRT) retention times is crucial to retain the biomass in the process.

The use of membrane filtration has been recommended because PNSB have been hypothesized to primarily grow in suspension for catching photons and to settle slowly (Chitapornpan et al., 2012). However, membranes are intended to separate biomass from the treated wastewater, but do not foster the formation of a good settling sludge. In the lab, MBRs are used to maintain biomass in suspension (van der Star et al., 2008). A centrifugation step is still needed after the membrane filtration to efficiently concentrate and harvest the PNSB biomass downstream. In this context, coagulation agents can also be used to help biomass aggregation. Nonetheless, alternatives to MBRs can lead to capital and operational savings, since membrane filtration and fouling relate to substantial pumping energy and maintenance costs besides the use of plastic materials.

Intensification of PNSB-based environmental biotechnologies should be targeted by enhancing the bioaggregation and biofilm-forming ability of the biomass. Although previous works have not tailored SBR regimes to this end (Chitapornpan et al., 2012; Fradinho et al., 2013), the application of substrate gradients via SBR operation can be efficient to stimulate microbial aggregation and biomass accumulation. Granulation of activated sludge biomasses in SBR systems has been the trigger of BNR process intensification (Pronk et al., 2015; Winkler et al., 2018; Aqeel et al., 2019). This should lead to an efficient S/L separation, resulting in lowering costs for downstream processing by potentially reducing the need for ultrafiltration and centrifugation to concentrate the biomass. A SBR design also offers operational flexibility (Morgenroth and Wilderer, 1998) to manipulate reactor cycles and loading rates. Although offering less surface-to-volume ratio, the use of simple stirred-tank designs in SBR application can in addition lead to simpler scale-up than flat-sheet, tubular, or membrane-based processes.

Here, we investigated the possibility to develop a mixed-culture biotechnology process based on the enrichment of a concentrated and well-settling PNSB biomass out of activated sludge in a stirred-tank photobioreactor operated under SBR regime and continuously irradiated with IR light. Conditions to enrich and maintain a PNSB mixed culture were elucidated at bench, along with microbial competition in the PNSB guild. Biomass growth, aggregation, and composition were analyzed along with volumetric rates of C-N-P removal. The here-examined microbial ecology insights and aggregation propensity of the PNSB guild can sustain the development of bioengineering strategies for mixed-culture process development in simple SBR design for wastewater treatment and resource recovery from aqueous nutrient streams.

## Materials and Methods

### Cultivation Medium

The cultivation medium was calculated based on stoichiometric requirements to sustain PNSB growth and complemented with other minerals adapted from Kaewsuk et al. (2010) to meet with C-N-P anabolic requirements of PNSB. The stock solution consisted of (per liter): 0.914 g of CH_3_COONa⋅3H_2_O, 0.014 g of KH_2_PO_4_, 0.021 g of K_2_HPO_4_, 0.229 g of NH_4_Cl, 0.200 g of MgSO_4_⋅7H_2_O, 0.200 g of NaCl, 0.050 g of CaCl_2_⋅2H_2_O, 0.100 g of yeast extract, 1 mL of vitamin solution, and 1 mL of trace metal solution. The vitamin solution contained (per liter) 200 mg of thiamine–HCl, 500 mg of niacin, 300 mg of ρ-amino-benzoic acid, 100 mg of pyridoxine–HCl, 50 mg of biotin, and 50 mg of vitamin B12. The trace metal solution contained (per liter) 1100 mg of EDTA–2Na⋅2H_2_O, 2000 mg of FeCl3⋅6H_2_O, 100 mg of ZnCl2, 64 mg of MnSO_4_⋅H_2_O, 100 mg of H_3_BO_3_, 100 mg of CoCl_2_⋅6H_2_O, 24 mg of Na_2_MoO_4_⋅2H_2_O, 16 mg of CuSO_4_⋅5H_2_O, 10 mg of NiCl_2_⋅6H_2_O, and 5 mg of NaSeO_3_. Carbon sources were separated from nitrogen and phosphate sources to avoid contaminations.

### Anaerobic Sequencing-Batch Photobioreactor Setup

**FIGURE 1:**
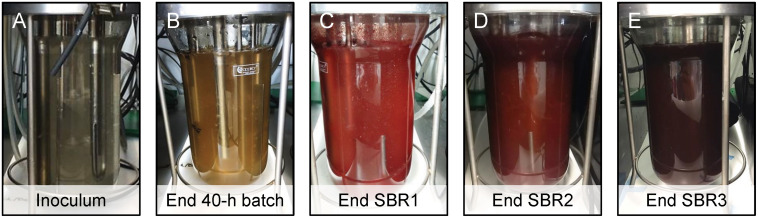
Anaerobic stirred-tank photobioreactor operated in sequencing batch mode to enrich for and aggregate PNSB. **(A)** The reactor was inoculated at 0.1 gVSS L^–1^ with BNR activated sludge. **(B)** One initial batch of 40 h was used to activate the sludge and test the enrichment of PNSB, prior to switching to SBR operations over 5 months. **(C)** *SBR1* was operated with 1 cycle d^–1^, HRT of 48 h, reaction phase of 20.75 h, and OLR of 0.215 g COD d^–1^ L_r_^–1^. **(D)** *SBR2* was operated with 3 cycles d^–1^, HRT of 16 h, reaction phase of 4.75 h, and OLR of 0.645 g COD d^–1^ L_r_^–1^. **(E)** *SBR3* was run like *SBR2* but fed at a doubled OLR of 1.290 g COD d^–1^ L_r_^–1^. The SRT was let freely evolve in the SBRs, increasing from 1.5 d (*SBR1*) to 7.2 d (*SBR2*) to 10.6 d (*SBR3*) along with PNSB aggregation and accumulation in the system from 0.1 (*SBR1*) to 1.6 (*SBR2*) to 3.0 (*SBR3*) g VSS L^–1^.

The PNSB enrichment was performed in a 1.5-L cylindrical, single-wall, glass, stirred-tank reactor (Applikon Biotechnology, Netherlands) (Figure 1). The reactor was inoculated at 0.1 g VSS L^–1^ of flocculent activated sludge taken from the BNR WWTP Harnaschpolder (the Netherlands) after washing the sludge with the cultivation medium three times (Figure 1A).

The reactor operated for 6 months under anaerobic SBR regime a temperature of 30 ± 1°C and pH of 7.0 ± 1.0 on an acetate-based synthetic wastewater. The temperature was controlled with a thermostat (WK 500, Lauda, Germany), providing coolant to a finger type heat exchanger (Applikon, Netherlands). The pH of the mixed liquor was controlled at 7.0 ± 1.0 by automatic addition of HCl or NaOH at 1 mol L^–1^ each. The bulk liquid was sparged with argon gas (quality 99.999%) to maintain anaerobic conditions, while continuously stirring at 378 rpm (potentiostat ADI 1012, Applikon, Netherlands) during the reaction phase.

To select for purple phototrophs and to avoid the proliferation of green phototrophs, the reactor was placed in a dark fume hood providing only IR light. A white light source was beamed with two halogen lamps (120 W, Breedstraler, GAMMA, Netherlands) placed at the side of the reactor and filtered for IR wavelengths (>700 nm) with two filter sheets (Black Perspex 962, Plasticstockist, United Kingdom) placed in front of the lamps. Irradiance was measured at the reactor surface with a pyranometer (CMP3, Kipp & Zonen, Netherlands) and set at a relatively high value of 375 W m^–2^ to promote PNSB enrichment and biomass growth. The light emission spectrum before and after the filter is provided in Supplementary Material 1.

### Sequencing-Batch Reactor Regimes

After a first 40-h period under batch regime to check for the selection of PNSB (Figure 1B), the system was switched to an SBR regime, consisting of discharge, idle, feed and settling phases. Different cycle timings and HRT, reaction phase length, and COD loading rates were tested as followed in three operational modes.

In *SBR1* (Figure 1C), 24 cycles of 24 h each were applied (i.e., 24 days of experiment), consisting of: biomass settling (3 h) effluent withdrawal (5 min), influent feeding (5 min), and reaction (20.75 h). In *SBR2* (Figure 1D), the total length of the cycles was decreased threefold and set at 8 h. The reaction phase was shortened to 4.75 h, while all the other phases were maintained. The reactor was operated over 205 cycles. In *SBR3* (Figure 1E), the cycle composition was maintained as in SBR2, while the COD concentration was doubled from 430 to 860 mg COD_Ac_ L^–1^ in the influent to prevent COD-limitations along the reaction phase.

All SBRs were operated at a volume exchange ratio of 50%. The stepwise adaptation of the SBR operations from 1 to 3 cycles day^–1^ resulted in HRTs from 48 h (SBR1) to 16 h (SBR2 and SBR3) and in volumetric organic loading rates (OLRs) of 0.215 (SBR1) to 0.645 (SBR2) and 1.290 (SBR3) kg COD d^–1^ m^–3^ (Table 1).

**TABLE 1:** Operational conditions of the three SBR regimes across the experimental period.

Process parameters	Units	SBR1	SBR2	SBR3
**SBR cycles**				
Number of SBR cycles per day	(–)	1	3	3
Reaction phase length per cycle	(h)	20.75	4.75	4.75
Discharge, idle, feeding phases lengths	(min)	5	5	5
Settling phase length	(h)	3	3	3
**Retention times**				
Hydraulic retention time (HRT)	(h)	48	16	16
Sludge retention time (SRT)^1^	(d)	1.5	7.2	10.6
**Loadings**				
Volumetric organic loading rate (OLR)	(g COD d^–1^ L_r_^–1^)	0.215	0.645	1.29
C:N:P ratio in the influent	(m/m/m)	100: 35.8: 3.8	100: 35.8: 4.2	100: 11.2: 1.7
**Measured initial concentrations in the bulk liquid phase at the beginning of reaction phases**				
Acetate	(mg COD L^–1^)	257 ± 54	232 ± 18	443 ± 76
Ammonium	(mg N-NH_4_^+^ L^–1^)	92 ± 21	83 ± 16	49 ± 17
Orthophosphate	(mg P-PO_4_^3+^ L^–1^)	9.7 ± 1.3	9.9 ± 3.3	7.7 ± 1.1

**FIGURE 2:**
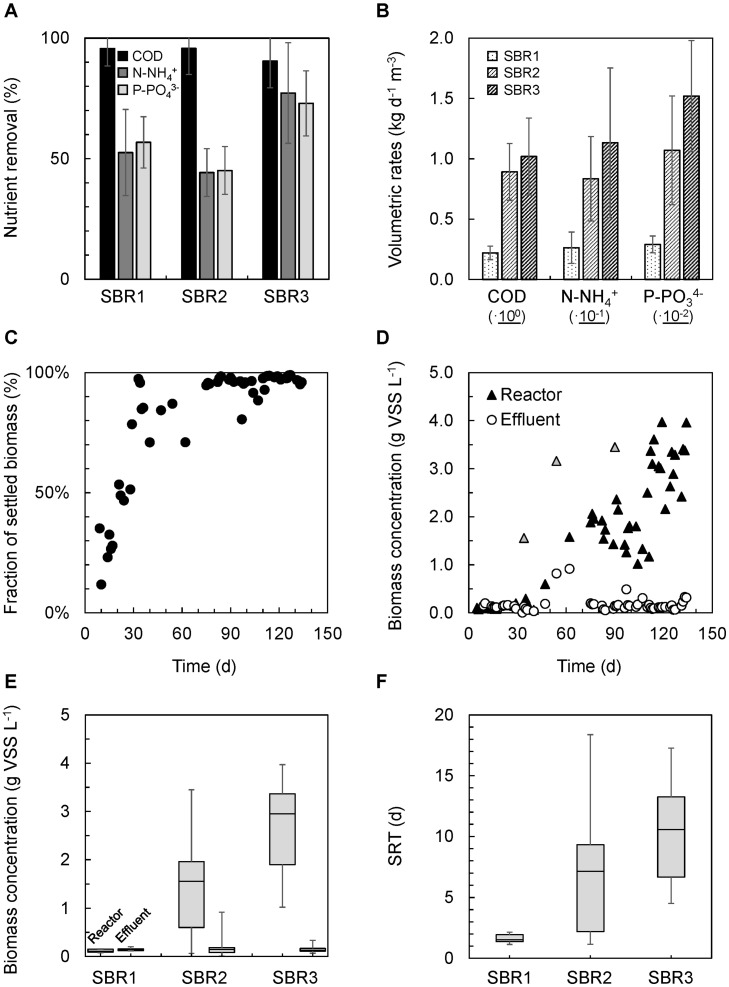
Nutrient removal and biomass characteristics across SBR operations in the mixed-culture PNSB photobioreactor. **(A)** Increases in COD, ammonium, and orthophosphate nutrient removal percentages from SBR1 to SBR3. On average, 95% of the COD was removed during all operational states. N-NH_4_^+^ and P-PO_4_^3–^ reached 77 and 73% of removal from the synthetic influent. **(B)** Gradual increases in volumetric rates of C-N-P nutrient removals from SBR1 to SBR3. **(C)** Increase in the fraction of mixed-liquor biomass that settled in the bioreactor along SBR1 (days 3–30), SBR2 (days 30–100), and SBR3 (days 100–135) after inoculation with BNR activated sludge and a first batch of 40 h. **(D)** Accumulation of biomass in the photobioreactor from SBR1 to SBR2 and SBR3. The gray triangles relate to cleanings and resuspensions of the wall biofilm in the biosystem. They indicate the total amount of biomass that accumulated in the reactor. **(E)** Distributions of biomass concentrations in the reactor at the end of the reaction phase and in the effluent after settling. The settling ability of the biomass increased steadily during SBR operations. The residual biomass concentration in suspension at the end of the settling phase in SBR3 was 10 times lower than the concentration in the mixed liquor during reaction time, displaying the well-settling property of the PNSB-enriched biomass. **(F)** The SRT was let freely evolve in the reactor, increasing from median values of 2 days (SBR1) to 7.5 days (SBR2) and 11 days (SBR3) along with biomass accumulation.

*SBR1 was run with a 24-h cycle composed of 20.75 h of reaction time and with 257 ± 54 mg COD L^–1^ in the bulk liquid phase after feeding. In SBR2, the total cycle length was shortened to 8 h, with a reaction time of 4.75 h. In SBR3, the initial COD concentration was doubled compared to the first two SBRs. All SBRs were run at a volume exchange ratio of 50%. ^1^The sludge retention time (SRT) was let freely evolve over the experimental period. The reactor was operated without purge of biomass. The SRT increased as a result of biomass accumulation in the reactor. The median value over each SBR period is provided. The distributions of SRT are displayed in Figure 2 and detailed evolutions in Supplementary Material 3.*

The SRT was let freely evolve across the SBR operations without controlled purge of the biomass. The measured SRTs resulting from biomass accumulation ranged from 1.5 to 11 days as median values calculated from eq. 2 in Supplementary Material 2.

### Analytical Methods to Measure Growth and Nutrient Consumptions

#### Measurements of Biomass Growth and Nutrient Concentrations

Biomass growth was monitored spectrophotometrically by absorbance at a wavelength of 660 nm (DR3900, Hach, Germany) 4–5 times a week (Supplementary Material 3), and gravimetrically by quantifying the concentration of volatile suspended solids (VSS) as described in experimental methods for wastewater treatment (van Loosdrecht et al., 2016). For the 40-h batch and SBR1, absorbance measurements were adequate since the biomass was low concentrated and in suspension. For SBR2 and SBR3, the biomass aggregated and VSS measurements were much more accurate.

The consumption of the dissolved nutrients was monitored by sampling the mixed liquor at the beginning and end of the reaction phase, after centrifugation (5 min, 17000 × *g*) and filtration of the supernatant on 0.45-μm filters (Millex-HV, PVDF, Germany). The concentrations of COD, ammonium (as N-NH_4_^+^) and orthophosphate (as P-PO_4_^3–^) were measured by colorimetric assays (LCK kits no. 114/614/COD, 302/303/ammonium, 348/350/phosphate; Hach-Lange, Dusseldorf, Germany) followed by spectrophotometry (DR3900, Hach, Germany). The COD colorimetric method measured all oxidizable substances (here notably acetate, yeast extract, and EDTA from the trace element solution). As technical control, samples were measured in triplicates and the relative standard deviation was 0.5 – 1.9%.

#### Computations of Microbial Conversions and Extraction of Growth Parameters

All symbols and equations used to compute microbial conversions and extraction of growth parameters are available in Supplementary Material 2.

In short, the average percentage of removal (η_S_,%), total rate of nutrient removal (R_S_, kg S d^–1^), apparent volumetric rate of removal of nutrients (r_S_, kg S d^–1^ m^–3^), and apparent growth rate (μ_max_, d^–1^) were calculated using mass balances over the C-N-P nutrients and biomass at a volumetric exchange ratio (VER) of 50%. Measurements of nutrients were performed at the beginning and end of the batch reaction phases of the SBR. Influent concentrations were back-calculated using the VER. The concentrations of nutrients in the effluent were assumed identical as at the end of the reaction phase.

Basic kinetic and stoichiometric parameters for microbial conversions and growth were assessed from nutrient consumptions and biomass production profiles using Aquasim (Reichert, 1994). A mathematical model was constructed using mass balances for substrate consumption and biomass production, and fitted to the experimental data (Supplementary Material 4). The maximum biomass-specific rate of acetate consumption (q_S,max_, kg S d^–1^ kg X), maximum yield of biomass production on substrate (Y_X/S,max_, kg X kg^–1^ S), and maintenance rate on substrate (m_S_, kg S d^–1^ kg X) were derived by parameter fit from the Herbert-Pirt relation of substrate allocation for growth. The biomass-specific maximum rate of growth (μ_max_, d^–1^) was computed from the relation between q_S,max_ and Y_X/S,max_, assuming the maintenance rate negligible versus the maximum growth rate during the exponential phase of the batch reaction period.

### Analysis of Biomass and Microbial Community Compositions

#### Light Microscopy Analysis of Microbial Morphotypes and Bioaggregates

Microbial morphotypes present in the enrichment were visually observed by phase contrast microscopy (Axioplan 2, Zeiss, Germany).

#### Biomass Settling Property

The fast biomass settling property achieved during the operation of SBR3 was characterized by calculation of the sedimentation G-flux of the solids (Driessens et al., 1987), as alternative to the sludge volume index (SVI) that was not measured. The G-flux was calculated based on the following formula (Eq. 1) derived in Supplementary Material 5.

(1) $$G-f\,l\,u\,x\,\left(k\,g\,V\,S\,S\,h^{-1}\,m^{-2}\right)=\frac{c_{X}\,\left(k\,g\,V\,S\,S\,m^{-3}\right)\cdot V_{R}\,\left(m^{3}\right)}{\Delta \,t_{s\,e\,t\,t\,l\,i\,n\,g}\,\left(h\right)\cdot \frac{\pi \cdot D\,{\left(m\right)}^{2}}{4}}$$

#### Wavelength Scan Analysis of Pigment Content in the PNSB-Enriched Biomass

The evolution of the biomass contents in bacteriochlorophyll a (BChl a) and carotenoids in the biomass was used as a proxy for tracking the PNSB enrichment in the mixed liquor. Measurements were performed by wavelength scan over the visible and near-infrared spectrum from 400 to 1000 nm (DR3900, Hach, Germany). A focus was attributed to absorbance peaks between 800–900 nm (BChl a) and 400–600 nm (carotenoids).

#### V3–V4 16S rRNA Gene Amplicon Sequencing of Bacterial Community Compositions

Genomic DNA was extracted from biomass samples throughout the duration of the experiment, using UltraClean Microbial Isolation kits (MOBIO laboratories, Inc., United States) following manufacturer’s instructions, and stored at −20°C. The concentrations and qualities of the DNA extracts were measured by Qbit3 fluorimeter (Thermofisher Scientific, United States), according to manufacturer’s instructions.

The DNA extracts were sent to Novogene (China) for amplicon sequencing. The V3–V4 regions of the 16S rRNA gene were amplified by polymerase chain reaction (PCR) using the set of forward V3-V4 forward 341f (5′-CCTACGGGAGGCAGCAG-3′) and reverse 806r (5′-GGACTACHVGGGTWTCTAAT-3′) primers (Takahashi et al., 2014). The amplicon sequencing libraries were pooled and sequenced paired-end in a MiSeq benchtop sequencer (Illumina).

After sequencing, the raw reads were quality filtered, chimeric sequences were removed, and OTUs were generated on the base of ≥97% identity. Subsequently, microbial community analysis was performed by Novogene using Mothur & Qiime software (V1.7.0). For phylogenetical determination the most recent SSURef database from SILVA^1^ was used. Relative abundances of OTUs were reported as % total sequencing reads count.

The sequences are deposited under NCBI Sequence Read Archive (SRA): BioProject ID PRJNA681757 (https://www.ncbi.nlm.nih.gov/bioproject/681757).

## Results

### High and Simultaneous Removal of C-N-P Nutrients Was Achieved in the PNSB-Enriched, Mixed-Culture, Stirred-Tank SBR

The enrichment grade of PNSB could be followed visually from the development of the purple color along the successive batch and SBR operations of the anaerobic stirred-tank photobioreactor inoculated with BNR activated sludge (Figures 1A–E). The nutrient removal performances achieved by the PNSB-based process from SBR1 to SBR2 and SBR3 regimes are displayed in Figure 2 and Table 2. The detailed dynamics in nutrient and biomass concentrations and compositions are provided in Supplementary Material 6.

**TABLE 2:** Nutrient removal by the PNSB-enriched biomass in SBR1, SBR2, and SBR3 presented as averages and maximal values of removal rates and removal percentages.

Chemical parameter	Units	SBR1	SBR2	SBR3
**Organic matter (as COD)**				
Volumetric removal rates	(kg d^–1^ m^–3^)	0.220 ± 0.056	0.891 ± 0.235	1.019 ± 0.318
Removal percentage	(%)	96 ± 7	96 ± 11	91 ± 11
Maximal volumetric removal rates	(kg d^–1^ m^–3^)	0.387	1.488	2.437
Maximal removal percentage	(%)	100	100	98
Average residual concentrations	(mg L^–1^)	67 ± 25	62 ± 32	61 ± 6
**Ammonium (as N-NH_4_^+^)**				
Volumetric removal rates	(g d^–1^ m^–3^)	26 ± 13	83 ± 35	113 ± 62
Removal percentage	(%)	53 ± 18	44 ± 10	77 ± 21
Maximal volumetric removal rates	(g d^–1^ m^–3^)	52	159	65
Maximal removal percentage	(%)	83	60	94
Average residual concentrations	(mg L^–1^)	39 ± 16	39 ± 6	11 ± 7
**Orthophosphate (as P-PO_4_^3–^)**				
Volumetric removal rates	(g d^–1^ m^–3^)	3 ± 1	11 ± 5	15 ± 5
Removal percentage	(%)	60 ± 11	45 ± 10	73 ± 14
Maximal volumetric removal rates	(g d^–1^ m^–3^)	5	18	25
Maximal removal percentage	(%)	91	59	95
Average residual concentrations	(mg L^–1^)	4 ± 1	5 ± 1	2 ± 1

*Residual concentration and removal percentages for COD met with European legislation limits for all SBRs (averages above 90% removal, with residues close to 60 mg COD L^–1^). Ammonium and orthophosphate were removed up to 77 and 73%, respectively, with residual concentrations reaching 11 mg N-NH_4_^+^ L^–1^ and 2 mg P-PO_4_^3–^ L^–1^.*

#### Nutrient Removing Activities Were Detected During the Initial Batch

During the first 40 h of batch used to activate the biomass, nutrients were removed at 98% COD (as acetate), 52% N-NH_4_^+^, and 60% P-PO_4_^3–^ (Figure 2A). These related to apparent volumetric removal rates of 0.190 ± 0.048 kg COD d^–1^ m^–3^, 21.5 ± 8.6 g N d^–1^ m^–3^, and 2.5 ± 0.5 mg P d^–1^ m^3^ (Figure 2B). The COD:N:P consumption ratio was 100:7.5:0.12 (m/m/m) in this batch.

#### A Complete Removal of Acetate Was Achieved Across All SBR Operation Modes

During SBR1, the average percentage of removal of the biodegradable COD was 96%, with an average volumetric consumption rate of 0.220 ± 0.060 kg COD d^–1^ m^–3^. During SBR2, 96% of the COD was removed as well at a fourfold higher rate of 0.891 ± 0.235 kg COD d^–1^ m^–3^. The carbon was fully removed over the first hour of the reaction phase, resulting in remaining 3.75 h of substrate limitation. During SBR3, the COD load in the influent was doubled, and as a result, no nutrient limitation occurred during the reaction phase. COD remained highly removed at 91%, with a volumetric removal rate of 1.08 ± 0.32 kg d^–1^ m^–3^.

#### A Maximum of 85% of Ammonium and 74% of Phosphate Was Removed From the Inflow

The ammonium removal rates increased from 26 ± 13 g N-NH_4_^+^ d^–1^ m^–3^ in SBR1 to 83.4 ± 35 g N d^–1^ m^–3^ during SBR2, and 113.3 ± 62 g N d^–1^ m^–3^ during SBR3. Average N-removal percentages evolved from 53 to 44 and 77% of the ammonium load across the three SBRs, respectively. Removal rates of orthophosphate increased from 3.0 ± 0.7 g P-PO_4_^3–^ d^–1^ m^–3^ of SBR1 to 10.7 ± 4.5 g P d^–1^ m^–3^ in SBR2 and 15.2 ± 4.6 g P d^–1^ m^–3^ in SBR3, with average P-removal percentages of 57, 45, and 73% per cycle, respectively. Under the non-limiting COD conditions of SBR3, the acetate, ammonium, and orthophosphate were released at median concentrations of 43 (min = 17; 1st–3rd quartile = 28–62) mg COD L_Eff_^–1^, 10 (2; 8–13) mg N-NH_4_^+^ L_Eff_^–1^, and 2.0 (0.4; 1.7–2.9) mg P-PO_4_^3–^ L_Eff_^–1^, i.e., close to European discharge criteria.

Thus, the average apparent COD:N:P assimilation ratio evolved from 100:7.5:0.12 in the batch to 100:9.2:1.2 (SBR1-2) under COD-limitation and 100:6.7:0.9 (SBR3) under non-COD-limitation. Across and beyond the experimental period, the intrinsic kinetics and stoichiometry of the PNSB-enriched biomass ranged with a biomass specific maximum growth rate (μ_max_) of 0.96–2.16 d^–1^ and a maximum yield of biomass production on substrate (Y_X/COD,max_) of 0.21–0.74 g VSS g^–1^ CODs, respectively. This related to a yield value of 0.34–1.19 g CODx g^–1^ CODs when using a theoretical elemental composition of C_1_H_1_._8_O_0_._38_N_0_._18_ (1.607 g CODx g^–1^ VSS) for purple phototrophic bacteria (Puyol et al., 2017a). The maximum biomass specific consumption of acetate (q_COD,max_) ranged from 0.03 to 0.78 kg CODs h^–1^ kg^–1^ VSS. These measurements were performed directly during SBR cycles at the actual concentration of the biomass present in the system. More accurate measurements and derivation of these physiological parameters can be performed at diluted initial concentrations of PNSB biomass to prevent nutrient and light limitations during batch tests.

#### Kinetic and Stoichiometric Parameters of Microbial Growth

The maximum biomass specific rate of substrate consumption (q_S__,max_), the yield of biomass growth of substrate consumption (Y_X/S_) and the maximum biomass specific growth rate (μ_max_) were obtained by parameter fit to batch evolutions of acetate and biomass during selected reaction phases of the SBRs (Table 3), using Aquasim (Supplementary Materials 4, 7).

**TABLE 3:** Observed physiological parameters of the biomass of the PNSB mixed culture extracted from the reaction periods of the initial batch and the three SBR periods, and comparison with literature data obtained from pure-culture and mixed-culture PNSB systems.

System	q_S,max_ (g COD_S_ d^–1^ g^–1^ VSS)	Y_X/S,max_ (g VSS g^–1^ COD_S_)	μ_max_ (d^–1^)
PNSB pure cultures	n.a.	0.98–1.23^a^	5.28^a^
PNSB mixed culture	n.a.	0.23–0.63^b^	0.72–1.68^b^
Initial batch	5.76	0.39	2.16
SBR1^c^	13.68 ± 5.04	0.23 ± 0.02	3.36 ± 1.4
SBR2^c^	1.76 ± 0.91	n.a. ^d^	n.a. ^d^
SBR3^c^	2.22 ± 0.79	n.a. ^d^	n.a. ^d^

*The values are given based on measured COD units for the acetate substrate and absorbance-calibrated VSS units for the biomass. The elemental formula for purple phototrophic bacteria C_1_H_1__.8_O_0__.38_N_0__.18_ (4.5 mol e^–^ C-mol^–1^, 36 g COD C-mol^–1^, 22.4 g VSS C-mol^–1^, 1.607 g COD_X_ g^–1^ VSS) (Puyol et al., 2017a) may be used for conversion of VSS in to COD units.^*a*^Taken from literature (Eroglu et al., 1999; Jih, 1998).^*b*^Taken from literature (Kaewsuk et al., 2010; Hülsen et al., 2014, 2016; Puyol et al., 2017a).^*c*^Average values collected over different cycles monitored over SBR1 (cycles 11, 17), SBR2 (cycles 21, 145, 166), and SBR3 (cycles 10, 31, 49, 91).^*d*^High biomass concentrations in the system. VSS and absorbance measurement are no sensitive enough to detect growth over reaction period. External batches with diluted biomass should be performed to this end.*

Under the conditions of the initial batch and of SBR1 (i.e., long HRT of 48 h, low OLR of 0.215 kg COD d^–1^ m^–3^, very low biomass concentration of 0.1 g VSS L^–1^, and low SRT of 1.5 days), the highly enriched PNSB biomass displayed high substrate consumption and growth rates. In the initial batch, q_S,max_ reached 5.8 g COD_S_ d^–1^ g^–1^ VSS and μ_max_ 2.2 d^–1^ (*N* = 1 model fit). For SBR1, q_S,max_ was computed as 13.7 ± 5.0 g COD_S_ d^–1^ g^–1^ VSS (average deviation; *N* = 2 model fits); the biomass maximized its growth rate with a substantial μ_max_ of 3.4 ± 1.4 d^–1^ (average deviation; *N* = 2) ranging between values reported in literature for mixed cultures and pure cultures of PNSB. The biomass in the batch and SBR1 thus developed at relatively low yields Y_X/S, max_ of 0.39 (*N* = 1) and 0.23 ± 0.02 g VSS g^–1^ COD_S_ (average deviation; *N* = 2), respectively. The maintenance rate (m_s_) was estimated to 0.72 g COD_S_ d^–1^ g^–1^ VSS.

Under the conditions of SBR2 and SBR3 (i.e., 3-times lower HRTs, 3–6-times higher OLRs, 16–30-times higher biomass concentrations, and 5–7-times longer SRTs), the biomass consumed acetate at a 3–8-fold lower q_S,max_ of 2.0 ± 0.8 g COD_S_ d^–1^ g^–1^ VSS (standard deviation; *N* = 7 model fits). The maximum growth rate and yield values could not be extracted from the data collected from the reactions phases of SBRs 2 and 3 at high biomass concentrations that link to nutrient limitations and low sensitivity of absorbance and VSS measurements to detect growth changes. To obtain these parameters and with accuracy, we recommend that batch tests should be conducted with a diluted biomass concentration either in a separate batch test vessel or directly in the reactor by using one small portion of the biomass while duly storing the full biomass to relaunch the SBR after the batch test.

### SBR Operations Enhanced the Settling Ability and Accumulation of the PNSB Biomass

The settling ability of the PNSB biomass increased across enrichment SBR operations, leading to substantial accumulation of biomass in the system from 0.1 (SBR1) to 1.6 (SBR2) to 3.0 (SBR3) g VSS L^–1^ as median values (Figures 2C,D). The enhancement of the settling ability was measured by comparing these biomass concentrations present in the mixed liquor at the end of the reaction phase with the concentrations in the effluent after the settling phase which was for all SBRs as low as 0.13–0.15 g VSS L_Eff_^–1^ (Figures 2C,D). The fraction of settled biomass increased across SBR1 from 12 to 53% of the VSS present in the mixed liquors at the end of reaction phases, reached 96% by end of SBR2, and remained high at 97 ± 3% over SBR3 (Figure 2C). The total rates of biomass accumulation calculated over the full settling period of 3 h increased from 0.02 ± 0.01 (SBR1) to 0.69 ± 0.46 (SBR2) and 1.30 ± 0.45 (SBR3) g VSS h^–1^, or from 0.02 ± 0.01 to 0.46 ± 0.31 and 0.87 ± 0.30 kg VSS h^–1^ m^–3^, respectively, when translated into volumetric rates. At the beginning of SBR1, the full 3 h period was required to settle the suspended biomass. At the end of SBR3, most of the 5.9 g VSS of biomass that aggregated and accumulated in the system settled in about 10 min (i.e., 35 gVSS h^–1^ or 24 kg VSS h^–1^ m^–3^ effectively). This high settling rate obtained on SBR3 corresponds to a sedimentation G-flux of the biomass solids of 4.5 kg VSS h^–1^ m^–2^ (Eq. 1 and Supplementary Material 5). This displayed the well-settling property of the aggregated PNSB biomass. It underlined potential for considerably shortening the settling phase and SBR cycle length in order to increase the daily loading of the system.

The fraction of VSS in the TSS remained relatively high with 85% (SBR1) to 93% (SBR2) to 80% (SBR3) as median values, i.e., corresponding to a fraction of inorganic suspended solids (ISS) between 7 and 20%. During SBR3 a period at lower VSS fraction with values below 60% and higher ISS fraction (>40%) was detected between days 97 and 117, underlying potential accumulation of inorganics, e.g., as intracellular polyphosphate (not measured), during nutrient assimilation in the biomass.

The SRT was let to increase freely, without controlled purge of biomass, as a result of the enhancement of settling properties of the biomass: it rose from 2 days in SBR1 to 7 days in SBR2 and 11 days in SBR3 as median values (Figure 2D). Strategies can be tested to control the SRT at specific values on the range between, e.g., 3–10 days, depending on nutrient capture and biomass production targets.

The PNSB enrichment process could be easily tracked visually with the gradual increase in the purple color intensity in the bioreactor (Figures 1A–D).

### Microscopy Images Showed an Increasing Size in Microbial Aggregates

**FIGURE 3:**
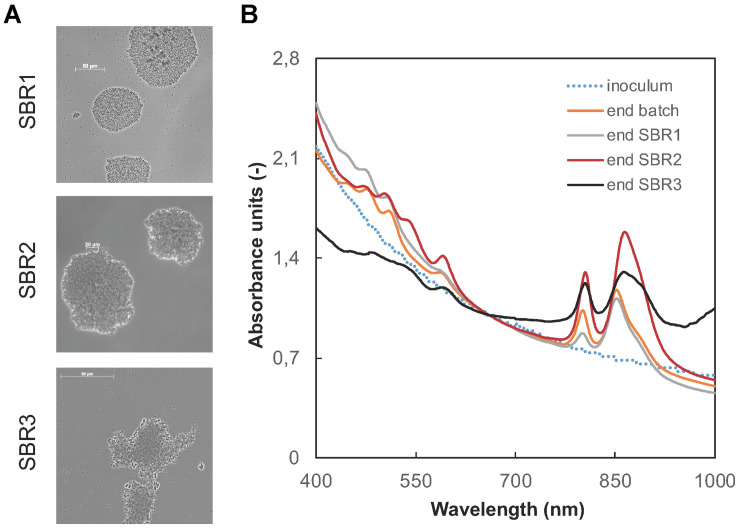
Evolution of the pigmentation and aggregative characteristics of the PNSB-enriched biomass. **(A)** Phase-contrast microscopy images of the aggregates present in SBR1 to SBR3. The size of the aggregates increased during time along with increased settling abilities of the biomass. **(B)** Wavelength scans of intact cultures, normalized for the biomass content (at 660 nm). The presence of PNSB was tracked at peaks around 800-900 nm (Bchl a) and 400–500 nm (carotenoids). After the initial batch phase of 40 h, the peaks typical for PNSB pigments were present, and persisted in the biomass until the end of SBR3.

After inoculation with flocculent activated sludge, phase-contrast microscopy imaging revealed the presence of dense aggregates already in SBR1 formed by the PNSB biomass (Figure 3A). Some cells clustered in flower-shaped aggregates, in a way comparable to the typical morphotype of *Rhodopseudomonas*. Other rod-shaped cells were present, putatively belonging to *Rhodobacter* and *Blastochloris* genera. The size of the aggregates increased from 50 to 150 μm during the operational time along with the better settling abilities of the biomass.

### Wavelength Scans Highlighted the Enrichment of Carotenoid and Bacteriochlorophyll Pigments in the Biomass and a Shift in Predominant Populations in the PNSB Guild

Carotenoids and bacteriochlorophylls, and their increase along the enrichment of the PNSB biomass, were detected by the presence of absorbance peaks at wavelengths between 450–500 nm and between 800–900 nm. The wavelength scan data presented in Figure 3B are normalized by the biomass concentrations, expressed as absorbance units at 660 nm. Peaks at 800 and 850 nm were already present at the end of the initial batch phase, and persisted during SBR1. At the end of SBR2, the absorbance peaks shifted to higher wavelengths of 805 and 865 nm. During SBR3, an increase in the absorbance was detected at 1000 nm. It is characteristic for the bacteriochlorophyll b, present in the genus *Blastochloris* but not in *Rhodopseudomonas* or *Rhodobacter*. This suggested a shift in predominant microbial populations harboring different types of pigments in the PNSB guild across the mixed-culture enrichment process.

### Amplicon Sequencing Revealed Selection Shifts From *Rhodobacter* to *Rhodopseudomonas* and *Blastochloris* Genera Within the Guild of PNSB

**FIGURE 4:**
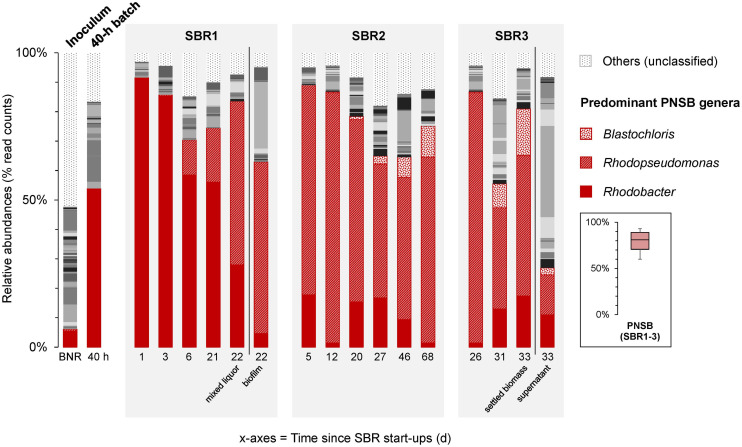
Time series of V3–V4 16S rRNA gene amplicon sequencing of bacterial community compositions in the PNSB-enriched mixed-culture process along SBR regime shifts. After inoculating the reactor with BNR activated sludge (“BNR”), a first PNSB genus *Rhodobacter* was initially enriched during the first 40-h batch (“40 h”) and early SBR1 period. The second PNSB genus *Rhodopseudomonas* was predominantly selected across operations of SBR2 and SBR3. The third PNSB genus *Blastochloris* popped up by end of SBR2 and SBR3. The PNSB guild remained predominant in the biomass across the process with an average total relative abundance of sequencing reads affiliated to known PNSB above 60% of the total community dataset (median = 81%; min-max = 60-93%). In SBR1, both the mixed liquor and the wall biofilm were sampled on day 22 and sequenced. In SBR3, both the settled biomass and s were sampled on day 33 after settling, and sequenced. The full set of genera is given in Supplementary Material 8.

The composition of the bacterial community of the mixed culture and underlying shifts in predominant populations were analyzed by V3–V4 16S rRNA gene amplicon sequencing. The times series of PNSB populations are displayed in Figure 4. The detailed times series of the full set of identified genera across the sequencing dataset is given in Supplementary Material 7.

The BNR activated sludge inoculum presented a diversity of genera, with *Rhodobacter* as the main PNSB detected at 4% of the sequencing read counts. The typical populations of the BNR sludge like ammonium oxidizer (*Nitrosomonas*), nitrite oxidizer (*Nitrospira*), denitrifier (*Zoogloea*), polyphosphate- (“*Candidatus* Accumulibacter”) and glycogen-accumulating (“*Ca.* Competibacter”) organisms got rapidly outcompeted right after start-up of the first batch under PNSB-selective conditions (Supplementary Material 7).

At the end of the 40-h batch phase, *Rhodobacter* reached a relative abundance of 52%. At the end of the first cycle of SBR1, a high-grade enrichment of 90% of *Rhodobacter* was obtained. Around the 10th cycle of SBR1 (10 days), the genus *Rhodopseudomonas* got enriched at 15%, and reached 50% at the end of the 23rd cycle (23 days after inoculum). The compositions of the communities of the mixed liquor and of the biofilm that developed on the walls of the reactor during the 13th cycle revealed that *Rhodopseudomonas* (55%) was outcompeting *Rhodobacter* (5%) in the biofilm, while *Rhodobacter* (60%) was more enriched than *Rhodopseudomonas* (10%) in the mixed liquor. Then, *Rhodobacter* decreased constantly from cycle to cycle, while *Rhodopseudomonas* progressively took the lead in the flocculent biomass as well.

After 18 cycles of SBR2 (i.e., 4.5 and 22.5 days from the starts of SBR2 and SBR1, respectively), *Rhodopseudomonas* became dominant (70%), outcompeting *Rhodobacter* (17%) in the enrichment culture. Interestingly, after 20 days of SBR2, the genus *Blastochloris*, also an affiliate of the PNSB guild, got selected, while the relative abundance of *Rhodopseudomonas* decreased to 60% at the end of SBR2. In SBR3, *Blastochloris* reached 10% of the bacterial community dataset.

## Discussion

### A High-Grade Enrichment of a Concentrated, Well-Settling PNSB Biomass Can Be Obtained Under SBR Regime

The enrichment of PNSB has often been successful, while most PNSB mixed cultures reported so far have mainly been in membrane systems (Hülsen et al., 2016). Here, we successfully enriched a mixed culture of PNSB out of activated sludge under traditional SBR regime in a stirred-tank system without the use of a membrane module to separate the biomass and the bulk liquid phase. This went by using the natural propensity of PNSB to form biofilms and bioaggregates. SBR regimes result in substrate gradients across reactor operation from high concentrations at the beginning of the cycle to low residual concentrations at the end. Such substrate gradients are known to promote the bioaggregation of microorganisms (Pronk et al., 2015; Winkler et al., 2018; Aqeel et al., 2019).

Promotion of bioaggregation of PNSB is key for a good S/L separation and accumulation of biomass in the system. One important outcome of this study highlighted that aggregation of PNSB can be stimulated under SBR regime to intensify the volumetric conversions and to facilitate downstream processing. After inoculation at 0.1 g VSS L^–1^, a high concentration of a PNSB-enriched biomass of up to a maximum of 4.0 g VSS L^–1^ was obtained in SBR3. The good settling ability of the PNSB biomass obtained under this regime resulted in the emission of less than 5% of the mixed liquor biomass in the effluent of SBR3, as low as 0.1 g VSS L^–1^. Interestingly, Driessens et al. (1987) have early reported the flocculation and good sedimentation (G-flux of 7–9 kg h^–1^ m^–2^ comparable to well-flocculated activated sludge) of *Rhodobacter capsulatus* in an upflow continuous photobioreactor operated under loading rates of 2.5–5.0 kg C d^–1^ m^–3^ (as calcium lactate; i.e., 6.7–13.3 kg COD d^–1^ m^–3^) and 0.5–1.0 kg N d^–1^ m^–3^ (as ammonium) with 87% C and N assimilation in the biomass (3.3–4.2 g VSS L^–1^). The PNSB-enriched biomass during SBR3 displayed a high sedimentation G-flux of 4.7 kg h^–1^ m^–2^ relatively close to the values reached by Driessens et al. (1987) under highly concentrated loading rates 5 to 10-fold higher than used here (max. 1.3 kg COD d^–1^ m^–3^ in SBR3). Collectively, this comparison sustains that PNSB can be aggregated for a higher accumulation and retention of biomass to intensify nutrient conversions.

In the PNSB mixed culture, the HRT was initially set high to 48 h (i.e., 1 cycle d^–1^ at a volume exchange ratio of 50%) to maintain biomass during start-up, prior to decreasing it to 16 h (3 cycles d^–1^) from SBR2 onward. This value was in the range of the HRTs of 8–24 h that have been used in the operation of continuous photo anaerobic membrane bioreactor (PAnMBR) to enrich for purple phototrophic bacteria (PPB) at bench (Hülsen et al., 2016). It was also in the range of traditional SBRs operated with conventional activated sludge (Mace and Mata-Alvarez, 2002). An operation at 4 cycles d^–1^ may be foreseen. Decreasing the settling phase length would lead to selectively retain the biomass fraction with higher settling property, with granulation potentialities. This is a typical approach to form a granular sludge out of flocculent activated sludge (de Kreuk and van Loosdrecht, 2006; Weissbrodt et al., 2013a; Lochmatter and Holliger, 2014; Winkler et al., 2018). Driving a granular sludge process with PNSB biomass can be of interest for process intensification.

The settling ability increased along the SBR operation, with a settled biomass fraction raising from 12% (SBR1) to 97% (SBR2-3). Amplicon sequencing revealed that the settled biomass accounted for a threefold higher relative abundance of PNSB (80% as sum of *Rhodobacter*, *Rhodopseudomonas*, and *Blastochloris*) than the non-settled biomass (25%) (Figure 4). Together with the phase-contrast microscopy measurements, this highlighted that PNSB are capable of forming bioaggregates with good settling properties. Such increased settling ability links to a more efficient separation of the PNSB biomass from the treated bulk liquid, thus facilitating the downstream processing to recover and valorize the PNSB biomass rich in nutrients for biorefinery purposes.

### A High, Simultaneous Removal of C-N-P Nutrients Was Achieved by the PNSB Biomass

High performances of organic matter (96% COD removal at a volumetric rate of 1.1 kg COD d^–1^ m^–3^), ammonium (77% N-removal at 113 g N d^–1^ m^–3^), and orthophosphate (73% P-removal at 15 g P d^–1^ m^–3^) removal were obtained under operation with a single anaerobic reaction phase using the PNSB process. Conventionally, a sequence of anaerobic, anoxic, and aerobic conditions is needed for full BNR in activated sludge or granular sludge (de Kreuk et al., 2005; Barnard and Abraham, 2006). The main difference relies that with PNSB single organisms can remove all nutrients by assimilation into the biomass by making use of photonic energy. BNR activated sludges make use of different microbial guilds of nitrifiers, denitrifiers, polyphosphate- and glycogen-accumulating organisms among others to remove all nutrients biologically. In activated sludge or granular sludge SBRs, the different redox conditions should be alternated to this end. Hence, this PNSB SBR process is a very interesting compact alternative to conventional BNR systems, that enables an enhanced removal of all nutrients in a single reaction phase by managing one single predominant microbial guild, thus simplifying considerably the microbial resource management.

With the PNSB biomass, the SBR process becomes simpler in terms of sequencing operation by feeding, anaerobic reaction, settling, and withdrawal. In practice, a fill/draw phase can be envisioned in function of the settling properties of the PNSB biomass. This can result in a SBR system operated by alternation of fill/draw and reaction phases only. Energy-wise, aeration is not needed in a PNSB process, resulting in possible electricity savings. In the case of sunlight use, electricity savings will be substantial. The tank will have to be equipped with light filters to supply IR light and select for PNSB as predominant phototrophs in the mixed culture. The irradiance of 375 W m^–2^ applied in this bench-scale photoSBR is high versus of practical operation window. Sunny regions of Europe are typically characterized by an annual average sunlight irradiance of 150 W m^–2^ (Posten, 2009). Nonetheless, light can be provided synthetically in photobioreactors using, e.g., immersed LED devices. In the case of ‘artificial’ supply of IR light, e.g., with LEDs, the process economics will have to be balanced with the electrical power needed to provide the irradiance needed to run the process. Biofilm formation on light-emitting tubes or light-emitting floating carriers will necessitate periodical cleaning to remediate shading, such as conventionally done for the maintenance of sensors used for process monitoring and control. The aim of this study was not to optimize the reactor design. Further thermoeconomical analysis will have to be conducted to determine the optimum irradiance to supply. This is analogical to the comparison of stirring performances in bench-scale reactors versus full-scale systems. There is room to study PNSB processes at different illumination intensities and their impact on the system responses such as enrichment grades, biomass concentrations, aggregation levels, and nutrient removal performances. Recent studies published on purple phototrophic bacteria have involved irradiances of *ca*. 50 W m^–2^ (Hülsen et al., 2016; Puyol et al., 2017a) which is about 8-times lower than the one used here at bench. However, no study has yet come with clear information on irradiance cutoffs and light patterns related to the microbial performance of PNSB in the mixed-culture and the economics of pilot and full-scale PNSB processes.

The volumetric removal rate of up to 1.1 kg COD d^–1^ m^–3^ achieved under the operation of SBR3 is comparable to the ranges of 0.8–2.5 kg COD d^–1^ m^–3^ reported for the PAnMBR (Hülsen et al., 2016), 0.2–1.4 kg COD d^–1^ m^–3^ for a continuous-flow stirred-tank reactor without separation of PNSB biomass (Alloul et al., 2019), and 1.2–3.2 kg COD d^–1^ m^–3^ for conventional BNR activated sludge processes (Tchobanoglous et al., 2003). It was nonetheless higher compared to aeration reactors, anaerobic ponds, and oxidation ditches (Tchobanoglous et al., 2003). Further enhancement of the COD loading rate and removal rate will be achieved by decreasing the SBR cycle time.

The difference in COD:N:P assimilation ratio between SBR1-2 (100:9.2:1.2 m/m/m) and SBR3 (100:6.7:0.9) resulted from the doubling of the acetate load in the influent. These COD:N:P assimilation ratios were in the range of ratios of 100:5.1-7.1:0.9-1.8 that have been characterized during growth of PPB (Hülsen et al., 2016; Puyol et al., 2017b). As comparison basis, a COD-N-P assimilation ratio of 100:5:1 is theoretically used for activated sludge (Henze et al., 2000).

The PNSB biomass of SBR3 harbored higher ISS content (>40%). Typical ISS fractions of 30–40% are widely detected in biomasses engineered for an enhanced biological phosphorus removal (EBPR) from wastewater (Weissbrodt et al., 2014). The reason for the higher ash fraction in the PNSB biomass not yet known but could be linked to mineralization, precipitation or intracellular polyphosphate storage processes among others. Future research should provide light on such abiotic or biotic processes in PNSB biomass. Besides, we showed that PNSB substantially remove COD, N and P up to more than 95, 80, and 70% respectively from the wastewater. Future ecophysiological elucidation of PNSB populations for the effective mechanism of phosphorus removal via anabolism only or polyphosphate uptake (Lai et al., 2017; Sakarika et al., 2020) or bio-induced precipitation will be of scientific and technological interests.

### Acetate and Wavelength Gradients Can Trigger Microbial Selection in the PNSB Guild

Phototrophic organisms are widespread in natural and anthropogenic environments. In the 16S rRNA gene amplicon sequencing analysis of the flocculating activated sludge here used as inoculum, around 4% of the total reads belonged to the genus *Rhodobacter*, and sequences corresponding to *Rhodopseudomonas* genus were also detected, highlighting the presence of PNSB in conventional sludges. Potentially, due to their versatile metabolism, different genera of PNSB can be constitutively present in activated sludges. Phototrophs derive the energy for their metabolism from the conversion of photons energy to chemical energy. The lack of requirements of substrate utilization to generate energy and external electron acceptors to catabolize acetate give to phototropic organisms a selective advantage over general chemotrophic organisms. PNSB absorb light in the IR spectrum to generate energy. The extensive IR irradiation along with a continuous stirring of the system led to the penetration of the light in the reactor, providing a selective pressure for the enrichment of PNSB. Spectrophotometric measurements of the biomass by wavelength scans from 300 to 900 nm revealed absorbance peaks characteristics for carotenoids and bacteriochlorophylls in PNSB. Peaks at 805 and 850 nm are typical for bacteriochlorophylls detected *in vivo* from cells of *Rhodopseudomonas capsulata* (Madigan and Gest, 1979), whereas a peak around 865 nm is typical for *Rhodobacter* (Zubova et al., 2005). These peaks were detected across the whole experimental period, indicating the presence and selection of PNSB organisms in the process. Pigments are excellent biomarkers of phototrophic populations, and provide specificity to distinguish between them (Stomp et al., 2007). Wavelength scan analyses are therefore very efficient for a rapid measurement (at min level) of the selection of PNSB.

The 16S rRNA gene amplicon sequencing analysis provided insights at higher resolution on the composition of the PNSB guild and underlying selection phenomena. Amplicon sequencing revealed a consistent enrichment of PNSB after already the first 40 h of batch. The initial enrichment of *Rhodobacter* followed by selection of *Rhodopseudomonas* and then *Blastochloris* can be explained by competition phenomena across substrate and wavelength gradients between these genera inside the guild of PNSB.

Okubo and Hiraishi (2007) have reported a preferential selection of *Rhodobacter* under high acetate concentration (5–20 mmol L^–1^, i.e., 320–1280 mg CODs L^–1^) due to its low affinity for acetate, while *Rhodopseudomonas* was enriched at lower concentrations (0.5 – 1 mmol L^–1^, i.e., 32–64 mg CODs L^–1^). The initial 40-h batch was fully loaded with acetate across the whole reaction period, making this condition favorable to select for *Rhodobacter*. Instead, *Rhodopseudomonas* harbors a higher affinity (i.e., lower affinity constant *K*_s_ of 0.11 mM for *Rhodopseudomonas* vs. 0.23 mM for *Rhodobacter*) (Okubo and Hiraishi, 2007) for acetate, enabling this population to grow more efficiently than *Rhodobacter* under acetate-limited conditions. During SBR1 and SBR2, the carbon source became progressively depleted after 1.5 h of reaction phase, leaving other 2.5 h of starvation period at low residual acetate concentration. This provided *Rhodopseudomonas* with a competitive advantage for growth.

The competition between *Rhodobacter* and *Rhodopseudomonas* may also be governed by their growth rate and thus the SRT in the system. Populations of *Rhodobacter* have displayed a higher maximum growth rate (1.8–2.2 d^–1^ in an enrichment and 2.3–3.8 d^–1^ with isolates) about 2.6 times faster than *Rhodopseudomonas* on VFA (Alloul et al., 2019). Batch regimes primarily select on growth rate: organisms deploy their maximum growth rate across most of a batch period during which substrate concentrations are mostly not limiting (Rombouts et al., 2019). The organism with the highest growth rate that can be activated under the actual operation conditions is therefore preferentially selected. This underlay the selection for *Rhodobacter* first prior to the establishment of *Rhodopseudomonas* along the progressive increase in SRT. Light availability can also be accounted as responsible for the selective enrichment of *Rhodobacter* or *Rhodopseudomonas*. As mentioned, bacteriochlorophylls from *Rhodobacter* and *Rhodopseudomonas* absorb at different wavelengths (800–850 vs. 865 nm respectively). At the beginning of the operation, the biomass concentration in the reactor was lower compared to following phases (i.e., SBR3). The initial higher availability of light at shorter wavelengths can have led to the selection of *Rhodobacter*, and similarly, the shadowing effect of the biomass can have acted as a natural filter, resulting in the selection of *Rhodopseudomonas*.

The genus *Blastochloris*, which appeared during SBR3, harbors bacteriochloropyll b (BChl b) instead of BChl a in *Rhodobacter* and *Rhodopseudomonas*. BChl b absorbs lower photonic energy at higher wavelengths (1020–1030 nm) (Hoogewerf et al., 2003) and can, therefore, interestingly survive at higher cell densities (here, around 1.5 g VSS L^–1^) with lower light penetration in the bulk liquid. Absorbance of the incident IR light increased across reactor operation with the development of a biofilm dominated by *Rhodopseudomonas* on the reactor wall and with a high concentration of PNSB biomass of up to 3.8 g VSS L^–1^ that accumulated in the reactor. According to the Beer–Lambert law, the accumulation of *Rhodobacter* and *Rhodopseudomonas* in the reactor and wall biofilm resulted in the absorbance of the higher-energy wavelengths in the 800–850 nm range of the IR light supplied, thus acting as wavelength filter. The lower-energy wavelengths not absorbed by *Rhodobacter* or *Rhodopseudomonas* were still available in the bulk-liquid, where *Blastochloris* could have absorbed them. The conjunction of the shading effect due to high biomass concentration and high SRT of 11 days were likely favorable for *Blastochloris* selection. Physiological characterization of this genus is needed in order to better predict its competition with other members of the diverse PNSB guild like *Rhodobacter* and *Rhodopseudomonas* among others. Light intensity and wavelengths are crucial parameters to consider for process development. Some studies evaluated these factors for biomass kinetics in PNSB pure cultures, but so far no literature is available for PNSB enrichments. Understanding the primers of the PNSB process ecology is of high interest for process design, management and control.

Overall, substrate gradients, light gradients, biofilm formation, and bioaggregation were identified as factors that triggered population selection and dynamics in the PNSB enrichment. Different lineages act in concert inside the guild of PNSB, providing metabolic redundancy and process resilience in the case of regime shifts in the process.

### PNSB Mixed Cultures From Bench Toward Process Development

The development of a lab-scale SBR system enriched for PNSB opens the doors for a possible upscaling of the process. A high nutrient capture was coupled with the production of a PNSB-rich biomass. Such biomass can be valorized for, e.g., proteins or PHAs productions (Honda et al., 2006; Alloul et al., 2018; Hülsen et al., 2018). The high settling ability of the biomass allows an easier solid-liquid separation of the latter from the treated water either in a compact external settler or directly in the SBR tank. This provides a downstream processing advantage over suspended biomass. It also overcomes the use of membrane filtration modules. The SBR regime resulted in the efficient aggregation of PNSB, underlying an enhanced settling ability and accumulation of biomass in the system. The SRT is an important process variable to control toward a stable bioprocess (Morgenroth and Wilderer, 1999). This becomes even more important in the perspective of harnessing the phosphorus removal capability of the PNSB biomass: cells saturated with phosphorus have to be effectively removed from the system such as conventionally performed by purge of excess sludge to maintain robust activated sludge or granular sludge processes operated for EBPR (Barnard and Abraham, 2006; Weissbrodt et al., 2013b). The growth rate and affinity for the substrate are further important parameter to manage the selection of PNSB populations in either batch or continuous-flow reactor regimes, respectively. Similarly, light irradiance is a key operational variable since it constitutes the primary energy source for PNSB. Light penetration and distribution are directly linked to the reactor geometry. In surface water ecosystems, IR light photons are typically consumed over the first 30 cm depth. Following the Beer–Lambert law, the absorbance of light will substantially increase with the biomass concentration. Shallow reactor systems can be opportune. SBR regimes can easily be transferred from stirred-tank to any reactor design, like raceway systems (or also known as carrousel plants) currently under investigation for green and purple phototrophic mixed-culture processes (Alloul et al., 2020). The application of substrate gradients via SBR or plug-flow reactor configurations can foster biomass aggregation to sustain efficient S/L separation for biomass recovery on top of nutrient capture.

## Conclusion

We investigated at bench the possibility to establish a mixed-culture PNSB process for nutrient capture from wastewater in an anaerobic photobioreactor operated as a traditional simple and flexible stirred-tank SBR. This work led to the following four main conclusions:

(1) SBR process conditions stimulated aggregation and accumulation (as high as 3.8 g VSS L^–1^) of a PNSB-enriched mixed culture in a fast-settling biomass that removed all nutrients biologically in a single reaction stage. The formation of compact aggregates facilitated S/L separation.
(2) Nutrient removal was substantial by assimilation in the biomass, reaching simultaneously 96% of organic matter at 1.1 kg COD d^–1^ m^–3^, 77% of ammonium at 113 g N d^–1^ m^–3^, and 73% of orthophosphate at 15 g P d^–1^ m^–3^, i.e., comparable to BNR activated sludge processes. Under non-COD-limiting conditions, the process reached the nutrient discharge limits set by the European Union.
(3) The PNSB guild accounted for as high as 90% of the bacterial community of the sludge (i.e., amplicon sequencing dataset), enabling a simple management of the microbial resource. A sequential selection between the genera *Rhodobacter*, *Rhodopseudomonas*, and *Blastochloris* was detected inside PNSB, allowing for functional redundancy in the microbiome and highlighting the microbial ecology of PNSB populations across light wavelengths.
(4) Next investigations should elucidate competition phenomena along growth rates, substrate affinities, and wavelength gradients across the mixed liquor.

For engineering practice, process analysis should cover the technological and economical aspects related to light supply in the bioreactor. The here-investigated biosystem was specifically designed for the enrichment and aggregation of PNSB thanks to an enhanced selective pressure under laboratory conditions. The applied restrictive conditions, such as IR light supply at high irradiance and argon bubbling to maintain anaerobic conditions, will have to be overcome to drive the applicability and economy of the process. The biological responses to light intensities, and the potential implications of high cell density in the reactor, have still to be evaluated to design an efficient and up-scalable process. Besides wastewater treatment, the value of the PNSB-based mixed-culture SBR process will reside in opportunities for water and resource recovery by valorization of the retained, concentrated, and nutrient-rich PNSB biomass.

## Data Availability Statement

The sequences are deposited under NCBI BioProject ID PRJNA681757 (https://www.ncbi.nlm.nih.gov/bioproject/681757).

## Author Contributions

DW, BS, and MC designed the study with contribution by SE and hints from AA. BS and MC performed the wet-lab experiments and measurements. MC, BS, and DW wrote the manuscript. MC and DW designed the artworks. SE, AA, and SV provided key inputs and feedbacks to the manuscript. All the authors read, revised, and accepted the manuscript.

## Conflict of Interest

The authors declare that the research was conducted in the absence of any commercial or financial relationships that could be construed as a potential conflict of interest.

## Footnotes

1 http://www.arb-silva.de/
