# Supplementary material for: Enrichment and Aggregation of Purple Non-sulfur Bacteria in a Mixed-Culture Sequencing-Batch Photobioreactor for Biological Nutrient Removal From Wastewater
Source: Front Bioeng Biotechnol. 2020 Dec 17;8:557234. doi: 10.3389/fbioe.2020.557234 (PMC7773948; doi:10.3389/fbioe.2020.557234)
Supplement: Supplementary file 1 [file Data_Sheet_1.pdf]

Supplementary material to:

# Enrichment and aggregation of purple non-sulfur bacteria in a mixed-culture sequencing-batch photobioreactor for biological nutrient removal from wastewater

Marta Cerruti[1,§], Berber Stevens[1,§], Sirous Ebrahimi[1,2], Abbas Alloul[3], Siegfried E. Vlaeminck[3], David G. Weissbrodt[1,§,*]

[1] Department of Biotechnology, Delft University of Technology, van der Maasweg 9, 2629 HZ Delft, Netherlands
[2] Department of Chemical Engineering, Sahand University of Technology, Tabriz, East Azerbaijan, Iran
[3] Department of Bioscience Engineering, University of Antwerp, Groenenborgerlaan 171, 2020 Antwerp, Belgium

[§] Equal contribution

*Correspondence: David Weissbrodt, Assistant Professor, Weissbrodt Group for Environmental Life Science Engineering, Environmental Biotechnology Section, Department of Biotechnology, Faculty of Applied Sciences, Delft University of Technology, van der Maasweg 9, 2629 HZ Delft, Netherlands; Phone: +31 15 27 81169; E-mail: d.g.weissbrodt@tudelft.nl.

## Supplementary material 1:

## Light spectrum profile of the halogen lamp with and without filter

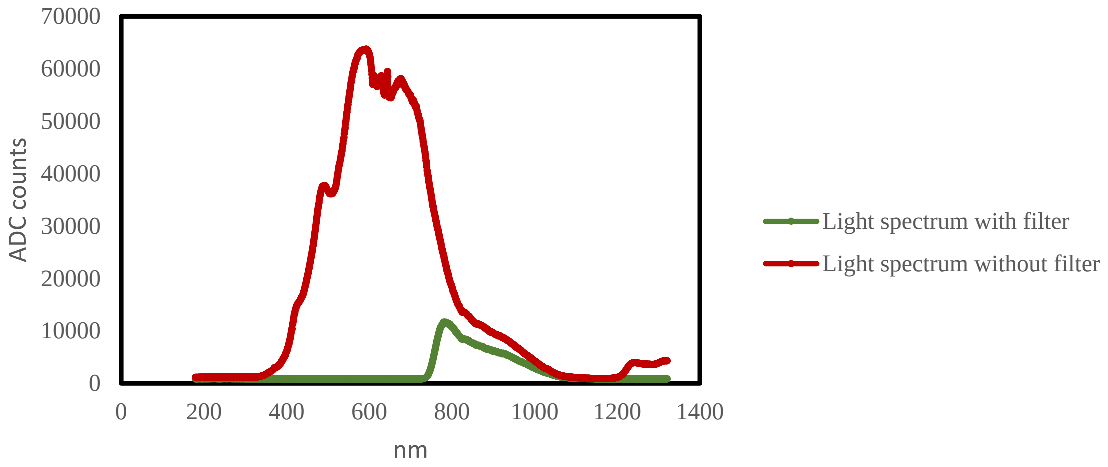


Figure SM1.1. The light spectrum was measured with high resolution fiber optic spectrometer AvaSpec-3648 (Avantes, the Netherlands) at a distance of 23 cm from the detector. The light spectrum was measured directly from the halogen lamp, and from the halogen lamp with the filter in front of it. A clear cut in the light emission is visible for the filtered light at wavelengths below 700 nm.

## Supplementary material 2:

## Symbols and Equations

Table SM2.1. Symbols used in calculation and model computation formula. Substrate concentrations were expressed in COD (organic matter), N-$NH_4^+$ (ammonium), and P-$PO_4^{3-}$ (orthophosphate) based units. Biomass concentrations were expressed in COD based units.

| Symbols | Units | Definition |
|---|---|---|
| $C_S$ | kg S $m^{-3}$ or mg S $L^{-1}$ | Concentration of substrate in the reactor (state variable) |
| $C_{S,inf}$ | kg S $m^{-3}$ or mg S $L^{-1}$ | Concentration of substrate in the influent |
| $C_{S,0}$ | kg S $m^{-3}$ or mg S $L^{-1}$ | Concentration of substrate at beginning of reaction phase |
| $C_{S,end}$ | kg S $m^{-3}$ or mg S $L^{-1}$ | Concentration of substrate at end of reaction phase |
| $C_{S,eff}$ | kg S $m^{-3}$ or mg S $L^{-1}$ | Concentration of substrate in the effluent |
| $C_X$ | kg X $m^{-3}$ or mg X $L^{-1}$ | Concentration of biomass in the reactor (state variable) |
| $C_{X,eff}$ | kg X $m^{-3}$ or mg X $L^{-1}$ | Concentration of biomass in the effluent |
| HRT | d or h | Hydraulic retention time |
| $K_S$ | kg S $m^{-3}$ or mg S $L^{-1}$ | Half-saturation affinity constant for substrate |
| $m_S$ | kg S $d^{-1}$ $kg^{-1}$ X | Biomass maintenance rate |
| $M_S$ | kg S | Mass of substrate |
| $M_X$ | kg X | Mass of biomass |
| $\mu$ or $q_X$ | kg X $d^{-1}$ $kg^{-1}$ X = $d^{-1}$ | Biomass specific growth rate |
| $N_{cycles}$ | cycles $d^{-1}$ | Number of SBR cycles per day |
| $\eta_S$ | % | Percentage of nutrient removal |
| $q_S$ | kg S $d^{-1}$ $kg^{-1}$ X | Biomass specific rate of substrate consumption |
| $Q_{inf} = Q_{eff} = Q$ | $m^3$ $cycle^{-1}$ or L $cycle^{-1}$ | Volume of influent fed and effluent withdrawn per SBR cycle |
| $Q_{sample}$ | $m^3$ $cycle^{-1}$ or mL $cycle^{-1}$ | Volume of mixed liquor samples collected during reaction phase |
| $Q_{purge}$ | $m^3$ $cycle^{-1}$ or mL $cycle^{-1}$ | Volume of mixed liquor purged at the end of reaction phase ($Q_{purge}$ = 0 if SRT let freely evolve) |
| $r_S$ | kg S $d^{-1}$ $m^{-3}$ | Apparent volumetric rate of nutrient removal |
| $R_S$ | kg S $d^{-1}$ | Total rate of nutrient removal |
| SRT | d | Sludge retention time |
| t | d or h | time |
| $t_{cycle}$ | h or d | SBR cycle time length |
| V | $m^3$ or L | Volume |
| $V_{inf}$ | $m^3$ or L | Volume of influent |
| $V_r$ | $m^3$ or L | Working volume of the reactor |
| $V_{eff}$ | $m^3$ or L | Volume of effluent |
| VER | % | Volume exchange ratio |

Table SM2.2. Equations used in calculations and model computations. Definitions and units of symbols are available in Table SM2.1 above.

Hydraulic retention time in a SBR, HRT (d or h)

$$HRT = \frac{V_r}{Q_{inf}\, N_{cycles}} \qquad \text{Eq. SM1}$$

Sludge retention time in a SBR, SRT (d)

$$SRT = \frac{C_x\, V_r}{(Q_{eff}\, C_{x,eff} + Q_{purge}\, C_x + Q_{sample}\, C_x)\, N_{cycles}} \qquad \text{Eq. SM2}$$

Apparent volumetric rate of nutrient removal, rS (kg S d$^{-1}$ m$^{-3}$)

$$rs = \frac{(Cs,\inf Qinf - Cs,eff\ Qeff)\ Ncycles}{Vr} = \frac{(Cs,\inf - Cs,eff)\ Q\ Ncycles}{Vr} \equiv \frac{Cs,0 - Cs,end}{tcycle} \qquad \text{Eq. SM3}$$

with:

$$Cs,0 = Cs,\inf\frac{Vinf}{Vr} + Cs,end\frac{Vr - Vinf}{Vr} = Cs,\inf VER - Cs,end\ (1 - VER)$$

$$Cs,inf = Cs,0\frac{1}{VER} - Cs,end\frac{1 - VER}{VER}$$

$$Cs,eff = Cs,end$$

$$VER = \frac{Vinf}{Vr} = \frac{Veff}{Vr}$$

Percentage of nutrient removal, ηS (%)

$$\eta s = \frac{Cs,inf - Cs,eff}{Cs,inf}100 = \left(1 - \frac{Cs,eff}{Cs,inf}\right)100 = \frac{Cs,0 - Cs,end}{Cs,0 - Cs,end\ (1 - VER)}100 \qquad \text{Eq. SM4}$$

Total rate of nutrient removal, RS (kg S d$^{-1}$)

$$|\ Rs\ | = \frac{dMs}{dt} = Vr\ \frac{dCs}{dt} = Vr\ rs \qquad \text{Eq. SM5}$$

Substrate consumption balance during a batch phase (at constant $V_r$)

$$\frac{dC_s}{d_t} = q_s\, C_x = q_{s,max}\, \frac{C_s}{C_s + K_s}\, C_x \qquad \text{Eq. SM6}$$

Biomass production balance during a batch phase (at constant $V_r$)

$$\frac{dC_x}{d_t} = \mu\, C_x = (q_s - m_s)\, Y_{sx,max}\ C_x = (q_{s,max}\, \frac{C_s}{C_s + K_s} - m_s\,)\, Y_{sx,max}\, C_x \qquad \text{Eq. SM7}$$

Herbert-Pirt equation for substrate allocation for growth and maintenance

Biomass specific rates of substrate consumption, $q_s$ (kg S d$^{-1}$ kg$^{-1}$ X)
Biomass specific growth rate, μ (kg X d$^{-1}$ kg$^{-1}$ X = d$^{-1}$)
Biomass maintenance rate, $m_s$ (kg S d$^{-1}$ kg$^{-1}$ X)

$$q_s = \frac{1}{Y_{x/s}}\mu + m_s \quad \text{or} \quad \mu = (q_s - m_s)\, Y_{x/s} \qquad \text{Eq. SM8}$$

$$\mu_{max} = \left(q_{s,max} - m_s\right) Y_{x/s} \approx q_{s,max}\, Y_{x/s} \quad \text{with } q_{s,max} \gg m_s \qquad \text{Eq. SM9}$$

## Supplementary material 3:

## Correlation between VSS and absorbance measurements

The correlation between the absorbance measurement and the VSS concentration of the PNSB-enriched biomass is described in Figure SM1.1 hereafter. It displays the experimental data of three measurement series with the linear regression line. Such a correlation analysis should checked on regular basis across a long-term experimental period in function of the composition of the biomass. It should also be done for every new experiment, since the content of pigments and of intracellular storage compounds of the PNSB biomass can vary from case to case, based on environmental conditions tested. Absorbance measurements of biomass are mainly applicable with biomass in suspension and at relatively low concentrations. It was mainly valid here for the first 40-h batch period and SBR1. As soon as biomass aggregates, traditional gravimetry measurements via TSS, ISS and VSS as described in standard methods are more effective. This was applied on SBR2 and SBR3. One should nonetheless keep in mind that gravimetry measurements do not differentiate cells and organic intracellular polymers such as PHAs.

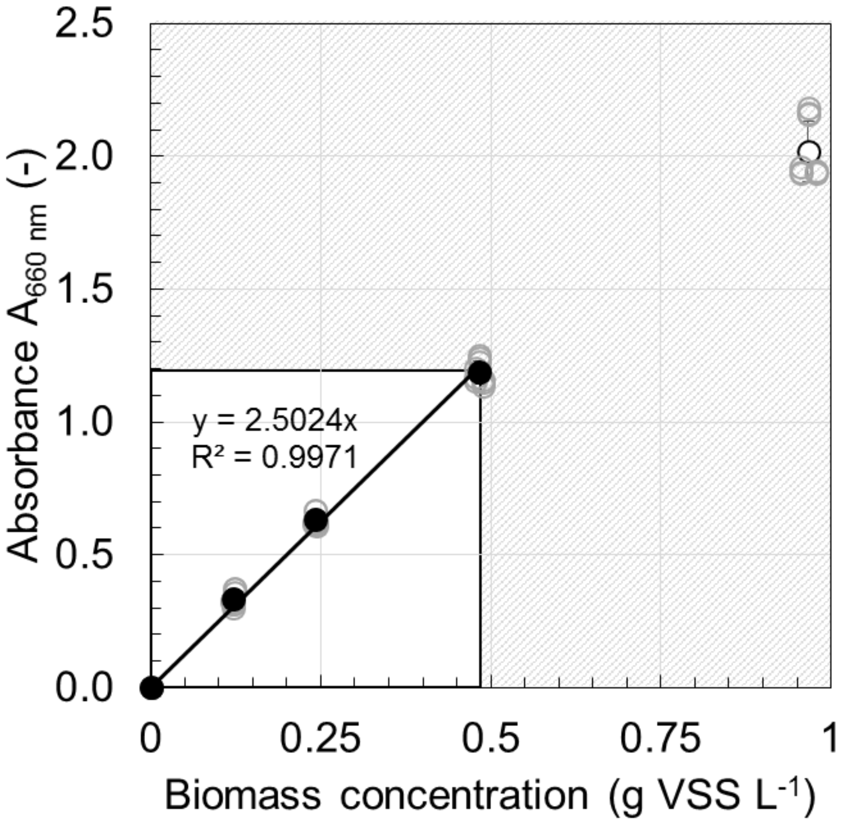


Figure SM3.1. Correlation between the absorbance at 660 nm and the VSS concentration of the PNSB-enriched biomass, based on dilution series of mixed liquor taken from the reactor (2×, 4×, 8×, 16× diluted). The linear regression of serie 1 is y = 0.3995x, serie 2 is y = 0.3856 x and serie 3 is y = 0.4124 x. For absorbance measurements, mixed-liquor samples were diluted to fit in the range from 0.2-1.2 absorbance units. On this abosrbance window the correlation to VSS was considered as linear. Absorbance data were mainly accurate measurements for the 40-h batch and SBR1 where the biomass was low concentrated and in suspension. In SBR2 and SBR3, the biomass aggregated and VSS measurements were much more accurate.

# Supplementary material 4:

# Aquasim model implementation

************************************************************************
AQUASIM Version 2.1f (win/mfc) - Listing of System Definition
************************************************************************

**Variables**
************************************************************************
------------------------------------------------------------------------
Cs: Description:
Type: Dyn. Volume State Var.
Unit:
Relative Accuracy: 1e-006
Absolute Accuracy: 1e-006
------------------------------------------------------------------------
Cs0: Description:
Type: Constant Variable
Unit:
Value: 200.83647
Standard Deviation: 6.3454465
Minimum: 0
Maximum: 1000
Sensitivity Analysis: inactive
Parameter Estimation: active
------------------------------------------------------------------------
Cs_exp: Description:
Type: Real List Variable
Unit:
Argument: t
Standard Deviations: global
Rel. Stand. Deviat.: 0
Abs. Stand. Deviat.: 1
Minimum: 0
Maximum: 1e+009
Interpolation Method: linear interpolation
Sensitivity Analysis: inactive
Real Data Pairs (14 pairs):
0 226
0.5 149.12
1 114.368
1.5 89.856
2 61.696
3 0
4 0
15 0
16 0
17 0
18 0
19 0
20 0
20.5 0
------------------------------------------------------------------------
Cx: Description:
Type: Dyn. Volume State Var.
Unit:
Relative Accuracy: 1e-006
Absolute Accuracy: 1e-006
------------------------------------------------------------------------
Cx0: Description:
Type: Constant Variable
Unit:
Value: 80.418816
Standard Deviation: 5.5701136

Minimum: 0
Maximum: 1000
Sensitivity Analysis: inactive
Parameter Estimation: active
------------------------------------------------------------------------
Cx_exp: Description:
Type: Real List Variable
Unit:
Argument: t
Standard Deviations: global
Rel. Stand. Deviat.: 0
Abs. Stand. Deviat.: 1
Minimum: 0
Maximum: 1e+009
Interpolation Method: linear interpolation
Sensitivity Analysis: inactive
Real Data Pairs (14 pairs):
0 73.902
0.5 91.869
1 94.92
1.5 103.056
2 115.599
3 129.837
4 133.566
15 112.209
16 111.192
17 111.531
18 112.548
19 108.819
20 110.175
20.5 117.633
------------------------------------------------------------------------
Ks: Description:
Type: Constant Variable
Unit:
Value: 1
Standard Deviation: 1
Minimum: 0
Maximum: 10
Sensitivity Analysis: inactive
Parameter Estimation: inactive
------------------------------------------------------------------------
ms: Description:
Type: Constant Variable
Unit:
Value: 0.034917139
Standard Deviation: 0.0099976129
Minimum: 0
Maximum: 10
Sensitivity Analysis: inactive
Parameter Estimation: active
Minimum: 0
Maximum: 1e+009
Interpolation Method: linear interpolation
Sensitivity Analysis: inactive
Real Data Pairs (4 pairs):
0 8.33
16 11.3
23 14.2
40 7.81
------------------------------------------------------------------------
qs_max: Description:
Type: Constant Variable
Unit:
Value: 0.7814633
Standard Deviation: 0.06061327
Minimum: 0

Maximum: 10
Sensitivity Analysis: inactive
Parameter Estimation: active
-----------------------------------------------------------------------
t: Description:
Type: Program Variable
Unit:
Reference to: Time
-----------------------------------------------------------------------
Ysx_max: Description:
Type: Constant Variable
Unit:
Value: 0.2513417
Standard Deviation: 0.047450747
Minimum: 0
Maximum: 10
Sensitivity Analysis: inactive
Parameter Estimation: active
************************************************************************

**Processes**
************************************************************************
Growth: Description:
Type: Dynamic Process
Rate: Cx*(qs_max*Cs/(Ks+Cs)-ms)*Ysx_max
Stoichiometry:
Variable : Stoichiometric Coefficient
Cx : 1
-----------------------------------------------------------------------
Substrate_uptake:
Description:
Type: Dynamic Process
Rate: Cx*qs_max*Cs/(Ks+Cs)
Stoichiometry:
Variable : Stoichiometric Coefficient
Cs : -1
************************************************************************

**Compartments**
************************************************************************
comp1: Description:
Type: Mixed Reactor Compartment
Compartment Index: 0
Active Variables: Cs, Cx
Active Processes: Growth, Substrate_uptake
Initial Conditions:
Variable(Zone) : Initial Condition
Cs(Bulk Volume) : Cs0
Cx(Bulk Volume) : Cx0
Inflow: 0
Loadings:
Volume: 1
Accuracies:
Rel. Acc. Q: 0.001
Abs. Acc. Q: 0.001
Rel. Acc. V: 0.001
Abs. Acc. V: 0.001
************************************************************************

**Definitions of Calculations**
*************************************************************************
calc1: Description:
Calculation Number: 0
Initial Time: 0
Initial State: given, made consistent
Step Size: 0.1
Num. Steps: 205
Status: active for simulation
inactive for sensitivity analysis
*************************************************************************

**Definitions of Parameter Estimation Calculations**
*************************************************************************
fit1: Description:
Calculation Number: 0
Initial Time: 0
Initial State: given, made consistent
Status: active
Fit Targets:
Data : Variable (Compartment,Zone,Time/Space)
Cs_exp : Cs (comp1,Bulk Volume,0)
Cx_exp : Cx (comp1,Bulk Volume,0)
*************************************************************************

**Plot Definitions**
*************************************************************************
plot1: Description:
Abscissa: Time
Title:
Abscissa Label:
Ordinate Label:
Curves:
Type : Variable [CalcNum,Comp.,Zone,Time/Space]
Value : Cs [0,comp1,Bulk Volume,0]
Value : Cx [0,comp1,Bulk Volume,0]
Value : Cs_exp [0,comp1,Bulk Volume,0]
Value : Cx_exp [0,comp1,Bulk Volume,0]
*************************************************************************

**Calculation Parameters**
*************************************************************************
Numerical Parameters: Maximum Int. Step Size: 1
Maximum Integrat. Order: 5
Number of Codiagonals: 1000
Maximum Number of Steps: 1000
----------------------------------------------------------------------
Fit Method: secant
Max. Number of Iterat.: 100
*************************************************************************

**Calculated States**
*************************************************************************
Calc. Num. Num. States Comments
0 14 Range of Times: 0 - 20.5
*************************************************************************

# Supplementary material 5:

# Calculation of the sedimentation G-flux of solids

G-flux (kg solids/h/$m^2$) calculated on SBR3 (fastest settling biomass)

Reactor working volume:

$V_R$ = 1.5 L = 0.0015 $m^3$

Reactor diameter:

$D_R$ = 10 cm = 0.1 m

Reactor cross section:

$A_R$ = $\pi \cdot D_R^2 / 4$ Eq. SM10

= $\pi \cdot 0.1^2 / 4 = 7.85 \cdot 10^{-3}$ $m^2$

Mass of biomass in system:

$m_X$ = $c_X \cdot V_R$ Eq. SM11

= 3.93 g VSS $L^{-1}$ · 1.5 L = 5.9 g VSS

Settling time achieved by the biomass in SBR3:

$\Delta t_{settling}$ = 10 min = 0.167 h

Total settling rate (TSR) of the biomass:

TSR = $m_X / \Delta t_{settling}$ Eq. SM12

= 5.9 g VSS / 0.167 h = 35.4 g VSS $h^{-1}$

Volumetric settling rate (VSR) of the biomass:

VSR = TSR / $V_R$ Eq. SM13

= 35.4 g VSS $h^{-1}$ / 1.5 L = 23.6 g VSS $h^{-1}$ $L^{-1}$ or kg VSS $h^{-1}$ $m^{-3}$

Sedimentation G-flux of the biomass:

**G-flux** = TSR / $A_R$ Eq. SM14

= 35.4 g VSS $h^{-1}$ / (7.85 · $10^{-3}$ $m^2$) = 4509 g VSS $h^{-1}$ $m^{-2}$

= **4.5 kg VSS $h^{-1}$ $m^{-2}$**

Generalized formula for G-flux: Eq. SM15

$$\text{G-flux (kg VSS h}^{-1}\text{ m}^{-2}\text{)} = \frac{c_X(\text{kg VSS m}^{-3}) \cdot V_R(\text{m}^3)}{\Delta t_{settling}(\text{h}) \cdot \frac{\pi \cdot D(\text{m})^2}{4}}$$

# Supplementary material 6:

## Dynamics of nutrient and biomass concentration and composition

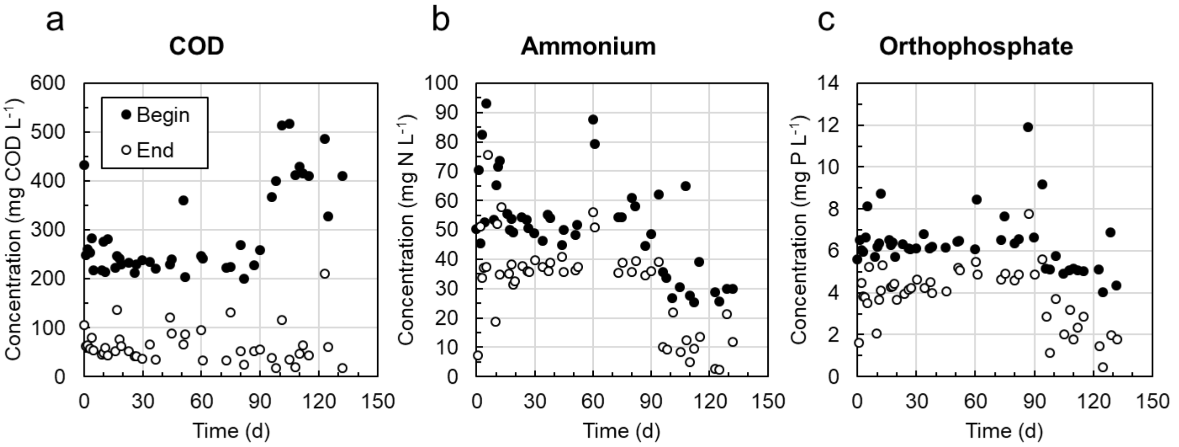


Figure SM6.1. Evolutions of COD (**A**), ammonium (**B**) and orthophosphate (**C**) removal across SBR1 (1st month), SBR2 (2nd-3rd months), SBR3 (4th-5th months). Concentrations at begin (*black dots*) and end (*white dots*) of the reaction phases of the SBRs are displayed. While acetate was fully removed, the remaining COD primarily related to EDTA present in the medium (*ca.* 50 mg COD $L^{-1}$).

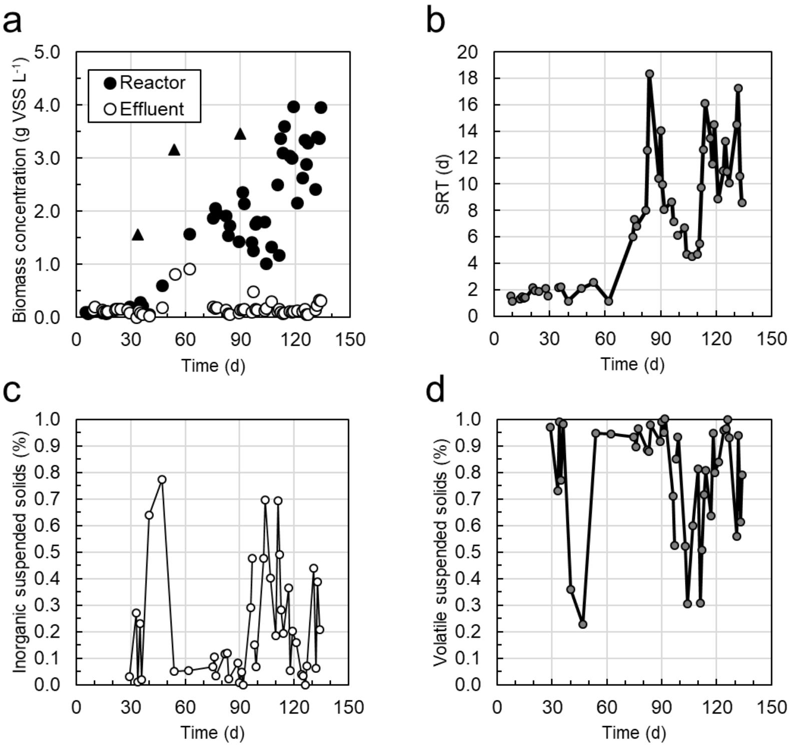


Figure SM6.2 Evolutions of (**A**) biomass concentrations in the reactor (*black dots*; *black triangles* relate to wall biofilm manual resuspension) and effluent (*white dots*); (**B**) sludge retention time (SRT); (**C-D**) fractions of inorganic (ISS) and volatile (VSS) suspended solids across SBR1 (1st month), SBR2 (2nd-3rd months), SBR3 (4th-5th months). The SRT was let freely evolve over the experimental period, without controlled purged of the mixed liquor.

## Supplementary material 7:

## Parameter fit in Aquasim along the 40-h batch and SBRs 1-3

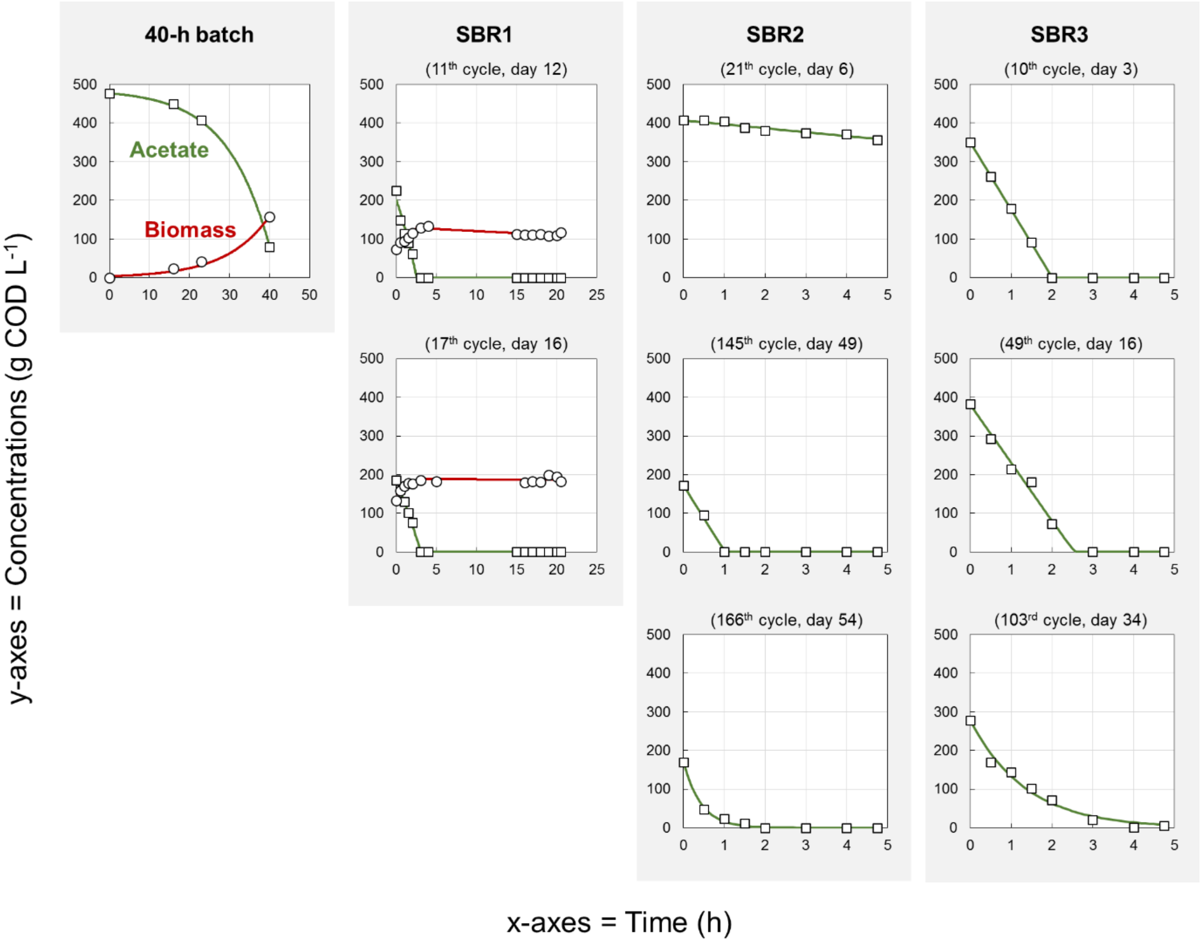


Figure SM7.1. Conversion dynamics during the 40-h batch and during the batch reaction phase of selected cycles in SBR1, SBR2, and SBR3 used for the computation of basic kinetic and stoichiometric parameters. Experimental data of biomass growth and COD depletion are displayed with the fitted Aquasim model along the batch reaction phase.

# Supplementary material 8:

## Detailed time series of V3-V4 16S rRNA gene amplicon sequencing

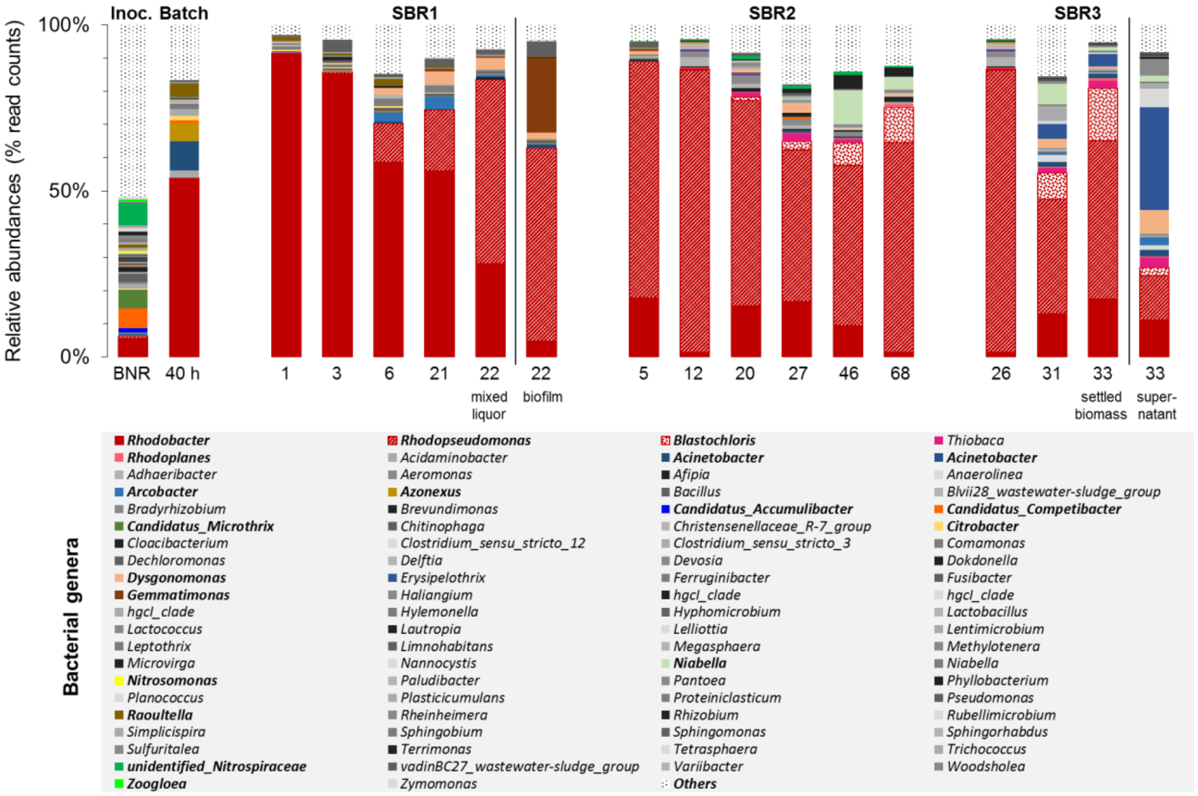


Figure SM8.1. Detailed time series of V3-V4 16S rRNA gene amplicon sequencing highlighting side populations evolving along the enrichment of PNSB in the mixed-culture process. The traditional BNR populations of the inoculum got rapidly outcompeted during the first batch after inoculation.
